# Measurement of prompt and nonprompt $\mathrm{J}/{\psi }$ production in $\mathrm {p}\mathrm {p}$ and $\mathrm {p}\mathrm {Pb}$ collisions at $\sqrt{s_{\mathrm {NN}}} =5.02\,\text {TeV}$

**DOI:** 10.1140/epjc/s10052-017-4828-3

**Published:** 2017-04-27

**Authors:** A. M. Sirunyan, A. Tumasyan, W. Adam, E. Asilar, T. Bergauer, J. Brandstetter, E. Brondolin, M. Dragicevic, J. Erö, M. Flechl, M. Friedl, R. Frühwirth, V. M. Ghete, C. Hartl, N. Hörmann, J. Hrubec, M. Jeitler, A. König, I. Krätschmer, D. Liko, T. Matsushita, I. Mikulec, D. Rabady, N. Rad, B. Rahbaran, H. Rohringer, J. Schieck, J. Strauss, W. Waltenberger, C.-E. Wulz, O. Dvornikov, V. Makarenko, V. Mossolov, J. Suarez Gonzalez, V. Zykunov, N. Shumeiko, S. Alderweireldt, E. A. De Wolf, X. Janssen, J. Lauwers, M. Van De Klundert, H. Van Haevermaet, P. Van Mechelen, N. Van Remortel, A. Van Spilbeeck, S. Abu Zeid, F. Blekman, J. D’Hondt, N. Daci, I. De Bruyn, K. Deroover, S. Lowette, S. Moortgat, L. Moreels, A. Olbrechts, Q. Python, K. Skovpen, S. Tavernier, W. Van Doninck, P. Van Mulders, I. Van Parijs, H. Brun, B. Clerbaux, G. De Lentdecker, H. Delannoy, G. Fasanella, L. Favart, R. Goldouzian, A. Grebenyuk, G. Karapostoli, T. Lenzi, A. Léonard, J. Luetic, T. Maerschalk, A. Marinov, A. Randle-conde, T. Seva, C. Vander Velde, P. Vanlaer, D. Vannerom, R. Yonamine, F. Zenoni, F. Zhang, A. Cimmino, T. Cornelis, D. Dobur, A. Fagot, M. Gul, I. Khvastunov, D. Poyraz, S. Salva, R. Schöfbeck, M. Tytgat, W. Van Driessche, E. Yazgan, N. Zaganidis, H. Bakhshiansohi, C. Beluffi, O. Bondu, S. Brochet, G. Bruno, A. Caudron, S. De Visscher, C. Delaere, M. Delcourt, B. Francois, A. Giammanco, A. Jafari, M. Komm, G. Krintiras, V. Lemaitre, A. Magitteri, A. Mertens, M. Musich, K. Piotrzkowski, L. Quertenmont, M. Selvaggi, M. Vidal Marono, S. Wertz, N. Beliy, W. L. Aldá Júnior, F. L. Alves, G. A. Alves, L. Brito, C. Hensel, A. Moraes, M. E. Pol, P. Rebello Teles, E. Belchior Batista Das Chagas, W. Carvalho, J. Chinellato, A. Custódio, E. M. Da Costa, G. G. Da Silveira, D. De Jesus Damiao, C. De Oliveira Martins, S. Fonseca De Souza, L. M. Huertas Guativa, H. Malbouisson, D. Matos Figueiredo, C. Mora Herrera, L. Mundim, H. Nogima, W. L. Prado Da Silva, A. Santoro, A. Sznajder, E. J. Tonelli Manganote, F. Torres Da Silva De Araujo, A. Vilela Pereira, S. Ahuja, C. A. Bernardes, S. Dogra, T. R. Fernandez Perez Tomei, E. M. Gregores, P. G. Mercadante, C. S. Moon, S. F. Novaes, Sandra S. Padula, D. Romero Abad, J. C. Ruiz Vargas, A. Aleksandrov, R. Hadjiiska, P. Iaydjiev, M. Rodozov, S. Stoykova, G. Sultanov, M. Vutova, A. Dimitrov, I. Glushkov, L. Litov, B. Pavlov, P. Petkov, W. Fang, M. Ahmad, J. G. Bian, G. M. Chen, H. S. Chen, M. Chen, Y. Chen, T. Cheng, C. H. Jiang, D. Leggat, Z. Liu, F. Romeo, M. Ruan, S. M. Shaheen, A. Spiezia, J. Tao, C. Wang, Z. Wang, H. Zhang, J. Zhao, Y. Ban, G. Chen, Q. Li, S. Liu, Y. Mao, S. J. Qian, D. Wang, Z. Xu, C. Avila, A. Cabrera, L. F. Chaparro Sierra, C. Florez, J. P. Gomez, C. F. González Hernández, J. D. Ruiz Alvarez, J. C. Sanabria, N. Godinovic, D. Lelas, I. Puljak, P. M. Ribeiro Cipriano, T. Sculac, Z. Antunovic, M. Kovac, V. Brigljevic, D. Ferencek, K. Kadija, B. Mesic, T. Susa, A. Attikis, G. Mavromanolakis, J. Mousa, C. Nicolaou, F. Ptochos, P. A. Razis, H. Rykaczewski, D. Tsiakkouri, M. Finger, M. Finger, E. Carrera Jarrin, Y. Assran, T. Elkafrawy, A. Mahrous, M. Kadastik, L. Perrini, M. Raidal, A. Tiko, C. Veelken, P. Eerola, J. Pekkanen, M. Voutilainen, J. Härkönen, T. Järvinen, V. Karimäki, R. Kinnunen, T. Lampén, K. Lassila-Perini, S. Lehti, T. Lindén, P. Luukka, J. Tuominiemi, E. Tuovinen, L. Wendland, J. Talvitie, T. Tuuva, M. Besancon, F. Couderc, M. Dejardin, D. Denegri, B. Fabbro, J. L. Faure, C. Favaro, F. Ferri, S. Ganjour, S. Ghosh, A. Givernaud, P. Gras, G. Hamel de Monchenault, P. Jarry, I. Kucher, E. Locci, M. Machet, J. Malcles, J. Rander, A. Rosowsky, M. Titov, A. Abdulsalam, I. Antropov, F. Arleo, S. Baffioni, F. Beaudette, P. Busson, L. Cadamuro, E. Chapon, C. Charlot, O. Davignon, R. Granier de Cassagnac, M. Jo, S. Lisniak, P. Miné, M. Nguyen, C. Ochando, G. Ortona, P. Paganini, P. Pigard, S. Regnard, R. Salerno, Y. Sirois, T. Strebler, Y. Yilmaz, A. Zabi, A. Zghiche, J.-L. Agram, J. Andrea, A. Aubin, D. Bloch, J.-M. Brom, M. Buttignol, E. C. Chabert, N. Chanon, C. Collard, E. Conte, X. Coubez, J.-C. Fontaine, D. Gelé, U. Goerlach, A.-C. Le Bihan, P. Van Hove, S. Gadrat, S. Beauceron, C. Bernet, G. Boudoul, C. A. Carrillo Montoya, R. Chierici, D. Contardo, B. Courbon, P. Depasse, H. El Mamouni, J. Fay, S. Gascon, M. Gouzevitch, G. Grenier, B. Ille, F. Lagarde, I. B. Laktineh, M. Lethuillier, L. Mirabito, A. L. Pequegnot, S. Perries, A. Popov, D. Sabes, V. Sordini, M. Vander Donckt, P. Verdier, S. Viret, A. Khvedelidze, Z. Tsamalaidze, C. Autermann, S. Beranek, L. Feld, M. K. Kiesel, K. Klein, M. Lipinski, M. Preuten, C. Schomakers, J. Schulz, T. Verlage, A. Albert, M. Brodski, E. Dietz-Laursonn, D. Duchardt, M. Endres, M. Erdmann, S. Erdweg, T. Esch, R. Fischer, A. Güth, M. Hamer, T. Hebbeker, C. Heidemann, K. Hoepfner, S. Knutzen, M. Merschmeyer, A. Meyer, P. Millet, S. Mukherjee, M. Olschewski, K. Padeken, T. Pook, M. Radziej, H. Reithler, M. Rieger, F. Scheuch, L. Sonnenschein, D. Teyssier, S. Thüer, V. Cherepanov, G. Flügge, B. Kargoll, T. Kress, A. Künsken, J. Lingemann, T. Müller, A. Nehrkorn, A. Nowack, C. Pistone, O. Pooth, A. Stahl, M. Aldaya Martin, T. Arndt, C. Asawatangtrakuldee, K. Beernaert, O. Behnke, U. Behrens, A. A. Bin Anuar, K. Borras, A. Campbell, P. Connor, C. Contreras-Campana, F. Costanza, C. Diez Pardos, G. Dolinska, G. Eckerlin, D. Eckstein, T. Eichhorn, E. Eren, E. Gallo, J. Garay Garcia, A. Geiser, A. Gizhko, J. M. Grados Luyando, A. Grohsjean, P. Gunnellini, A. Harb, J. Hauk, M. Hempel, H. Jung, A. Kalogeropoulos, O. Karacheban, M. Kasemann, J. Keaveney, C. Kleinwort, I. Korol, D. Krücker, W. Lange, A. Lelek, T. Lenz, J. Leonard, K. Lipka, A. Lobanov, W. Lohmann, R. Mankel, I.-A. Melzer-Pellmann, A. B. Meyer, G. Mittag, J. Mnich, A. Mussgiller, D. Pitzl, R. Placakyte, A. Raspereza, B. Roland, M. Ö. Sahin, P. Saxena, T. Schoerner-Sadenius, S. Spannagel, N. Stefaniuk, G. P. Van Onsem, R. Walsh, C. Wissing, V. Blobel, M. Centis Vignali, A. R. Draeger, T. Dreyer, E. Garutti, D. Gonzalez, J. Haller, M. Hoffmann, A. Junkes, R. Klanner, R. Kogler, N. Kovalchuk, T. Lapsien, I. Marchesini, D. Marconi, M. Meyer, M. Niedziela, D. Nowatschin, F. Pantaleo, T. Peiffer, A. Perieanu, J. Poehlsen, C. Scharf, P. Schleper, A. Schmidt, S. Schumann, J. Schwandt, H. Stadie, G. Steinbrück, F. M. Stober, M. Stöver, H. Tholen, D. Troendle, E. Usai, L. Vanelderen, A. Vanhoefer, B. Vormwald, M. Akbiyik, C. Barth, S. Baur, C. Baus, J. Berger, E. Butz, R. Caspart, T. Chwalek, F. Colombo, W. De Boer, A. Dierlamm, S. Fink, B. Freund, R. Friese, M. Giffels, A. Gilbert, P. Goldenzweig, D. Haitz, F. Hartmann, S. M. Heindl, U. Husemann, I. Katkov, S. Kudella, H. Mildner, M. U. Mozer, Th. Müller, M. Plagge, G. Quast, K. Rabbertz, S. Röcker, F. Roscher, M. Schröder, I. Shvetsov, G. Sieber, H. J. Simonis, R. Ulrich, S. Wayand, M. Weber, T. Weiler, S. Williamson, C. Wöhrmann, R. Wolf, G. Anagnostou, G. Daskalakis, T. Geralis, V. A. Giakoumopoulou, A. Kyriakis, D. Loukas, I. Topsis-Giotis, S. Kesisoglou, A. Panagiotou, N. Saoulidou, E. Tziaferi, I. Evangelou, G. Flouris, C. Foudas, P. Kokkas, N. Loukas, N. Manthos, I. Papadopoulos, E. Paradas, N. Filipovic, G. Pasztor, G. Bencze, C. Hajdu, D. Horvath, F. Sikler, V. Veszpremi, G. Vesztergombi, A. J. Zsigmond, N. Beni, S. Czellar, J. Karancsi, A. Makovec, J. Molnar, Z. Szillasi, M. Bartók, P. Raics, Z. L. Trocsanyi, B. Ujvari, J. R. Komaragiri, S. Bahinipati, S. Bhowmik, S. Choudhury, P. Mal, K. Mandal, A. Nayak, D. K. Sahoo, N. Sahoo, S. K. Swain, S. Bansal, S. B. Beri, V. Bhatnagar, R. Chawla, U. Bhawandeep, A. K. Kalsi, A. Kaur, M. Kaur, R. Kumar, P. Kumari, A. Mehta, M. Mittal, J. B. Singh, G. Walia, Ashok Kumar, A. Bhardwaj, B. C. Choudhary, R. B. Garg, S. Keshri, S. Malhotra, M. Naimuddin, K. Ranjan, R. Sharma, V. Sharma, R. Bhattacharya, S. Bhattacharya, K. Chatterjee, S. Dey, S. Dutt, S. Dutta, S. Ghosh, N. Majumdar, A. Modak, K. Mondal, S. Mukhopadhyay, S. Nandan, A. Purohit, A. Roy, D. Roy, S. Roy Chowdhury, S. Sarkar, M. Sharan, S. Thakur, P. K. Behera, R. Chudasama, D. Dutta, V. Jha, V. Kumar, A. K. Mohanty, P. K. Netrakanti, L. M. Pant, P. Shukla, A. Topkar, T. Aziz, S. Dugad, G. Kole, B. Mahakud, S. Mitra, G. B. Mohanty, B. Parida, N. Sur, B. Sutar, S. Banerjee, R. K. Dewanjee, S. Ganguly, M. Guchait, Sa. Jain, S. Kumar, M. Maity, G. Majumder, K. Mazumdar, T. Sarkar, N. Wickramage, S. Chauhan, S. Dube, V. Hegde, A. Kapoor, K. Kothekar, S. Pandey, A. Rane, S. Sharma, S. Chenarani, E. Eskandari Tadavani, S. M. Etesami, M. Khakzad, M. Mohammadi Najafabadi, M. Naseri, S. Paktinat Mehdiabadi, F. Rezaei Hosseinabadi, B. Safarzadeh, M. Zeinali, M. Felcini, M. Grunewald, M. Abbrescia, C. Calabria, C. Caputo, A. Colaleo, D. Creanza, L. Cristella, N. De Filippis, M. De Palma, L. Fiore, G. Iaselli, G. Maggi, M. Maggi, G. Miniello, S. My, S. Nuzzo, A. Pompili, G. Pugliese, R. Radogna, A. Ranieri, G. Selvaggi, A. Sharma, L. Silvestris, R. Venditti, P. Verwilligen, G. Abbiendi, C. Battilana, D. Bonacorsi, S. Braibant-Giacomelli, L. Brigliadori, R. Campanini, P. Capiluppi, A. Castro, F. R. Cavallo, S. S. Chhibra, G. Codispoti, M. Cuffiani, G. M. Dallavalle, F. Fabbri, A. Fanfani, D. Fasanella, P. Giacomelli, C. Grandi, L. Guiducci, S. Marcellini, G. Masetti, A. Montanari, F. L. Navarria, A. Perrotta, A. M. Rossi, T. Rovelli, G. P. Siroli, N. Tosi, S. Albergo, S. Costa, A. Di Mattia, F. Giordano, R. Potenza, A. Tricomi, C. Tuve, G. Barbagli, V. Ciulli, C. Civinini, R. D’Alessandro, E. Focardi, P. Lenzi, M. Meschini, S. Paoletti, L. Russo, G. Sguazzoni, D. Strom, L. Viliani, L. Benussi, S. Bianco, F. Fabbri, D. Piccolo, F. Primavera, V. Calvelli, F. Ferro, M. R. Monge, E. Robutti, S. Tosi, L. Brianza, F. Brivio, V. Ciriolo, M. E. Dinardo, S. Fiorendi, S. Gennai, A. Ghezzi, P. Govoni, M. Malberti, S. Malvezzi, R. A. Manzoni, D. Menasce, L. Moroni, M. Paganoni, D. Pedrini, S. Pigazzini, S. Ragazzi, T. Tabarelli de Fatis, S. Buontempo, N. Cavallo, G. De Nardo, S. Di Guida, M. Esposito, F. Fabozzi, F. Fienga, A. O. M. Iorio, G. Lanza, L. Lista, S. Meola, P. Paolucci, C. Sciacca, F. Thyssen, P. Azzi, N. Bacchetta, L. Benato, A. Boletti, R. Carlin, P. Checchia, M. Dall’Osso, P. De Castro Manzano, T. Dorigo, U. Dosselli, F. Gasparini, U. Gasparini, A. Gozzelino, S. Lacaprara, M. Margoni, A. T. Meneguzzo, J. Pazzini, M. Pegoraro, N. Pozzobon, P. Ronchese, M. Sgaravatto, F. Simonetto, E. Torassa, S. Ventura, M. Zanetti, P. Zotto, A. Braghieri, F. Fallavollita, A. Magnani, P. Montagna, S. P. Ratti, V. Re, C. Riccardi, P. Salvini, I. Vai, P. Vitulo, L. Alunni Solestizi, G. M. Bilei, D. Ciangottini, L. Fanò, P. Lariccia, R. Leonardi, G. Mantovani, M. Menichelli, A. Saha, A. Santocchia, K. Androsov, P. Azzurri, G. Bagliesi, J. Bernardini, T. Boccali, R. Castaldi, M. A. Ciocci, R. Dell’Orso, S. Donato, G. Fedi, A. Giassi, M. T. Grippo, F. Ligabue, T. Lomtadze, L. Martini, A. Messineo, F. Palla, A. Rizzi, A. Savoy-Navarro, P. Spagnolo, R. Tenchini, G. Tonelli, A. Venturi, P. G. Verdini, L. Barone, F. Cavallari, M. Cipriani, D. Del Re, M. Diemoz, S. Gelli, E. Longo, F. Margaroli, B. Marzocchi, P. Meridiani, G. Organtini, R. Paramatti, F. Preiato, S. Rahatlou, C. Rovelli, F. Santanastasio, N. Amapane, R. Arcidiacono, S. Argiro, M. Arneodo, N. Bartosik, R. Bellan, C. Biino, N. Cartiglia, F. Cenna, M. Costa, R. Covarelli, A. Degano, N. Demaria, L. Finco, B. Kiani, C. Mariotti, S. Maselli, E. Migliore, V. Monaco, E. Monteil, M. Monteno, M. M. Obertino, L. Pacher, N. Pastrone, M. Pelliccioni, G. L. Pinna Angioni, F. Ravera, A. Romero, M. Ruspa, R. Sacchi, K. Shchelina, V. Sola, A. Solano, A. Staiano, P. Traczyk, S. Belforte, M. Casarsa, F. Cossutti, G. Della Ricca, A. Zanetti, D. H. Kim, G. N. Kim, M. S. Kim, S. Lee, S. W. Lee, Y. D. Oh, S. Sekmen, D. C. Son, Y. C. Yang, A. Lee, H. Kim, J. A. Brochero Cifuentes, T. J. Kim, S. Cho, S. Choi, Y. Go, D. Gyun, S. Ha, B. Hong, Y. Jo, Y. Kim, K. Lee, K. S. Lee, S. Lee, J. Lim, S. K. Park, Y. Roh, J. Almond, J. Kim, H. Lee, S. B. Oh, B. C. Radburn-Smith, S. H. Seo, U. K. Yang, H. D. Yoo, G. B. Yu, M. Choi, H. Kim, J. H. Kim, J. S. H. Lee, I. C. Park, G. Ryu, M. S. Ryu, Y. Choi, J. Goh, C. Hwang, J. Lee, I. Yu, V. Dudenas, A. Juodagalvis, J. Vaitkus, I. Ahmed, Z. A. Ibrahim, M. A. B. Md Ali, F. Mohamad Idris, W. A. T. Wan Abdullah, M. N. Yusli, Z. Zolkapli, H. Castilla-Valdez, E. De La Cruz-Burelo, I. Heredia-De La Cruz, A. Hernandez-Almada, R. Lopez-Fernandez, R. Magaña Villalba, J. Mejia Guisao, A. Sanchez-Hernandez, S. Carrillo Moreno, C. Oropeza Barrera, F. Vazquez Valencia, S. Carpinteyro, I. Pedraza, H. A. Salazar Ibarguen, C. Uribe Estrada, A. Morelos Pineda, D. Krofcheck, P. H. Butler, A. Ahmad, M. Ahmad, Q. Hassan, H. R. Hoorani, W. A. Khan, A. Saddique, M. A. Shah, M. Shoaib, M. Waqas, H. Bialkowska, M. Bluj, B. Boimska, T. Frueboes, M. Górski, M. Kazana, K. Nawrocki, K. Romanowska-Rybinska, M. Szleper, P. Zalewski, K. Bunkowski, A. Byszuk, K. Doroba, A. Kalinowski, M. Konecki, J. Krolikowski, M. Misiura, M. Olszewski, M. Walczak, P. Bargassa, C. Beirão Da Cruz E Silva, B. Calpas, A. Di Francesco, P. Faccioli, P. G. Ferreira Parracho, M. Gallinaro, J. Hollar, N. Leonardo, L. Lloret Iglesias, M. V. Nemallapudi, J. Rodrigues Antunes, J. Seixas, O. Toldaiev, D. Vadruccio, J. Varela, P. Vischia, S. Afanasiev, P. Bunin, M. Gavrilenko, I. Golutvin, I. Gorbunov, A. Kamenev, V. Karjavin, A. Lanev, A. Malakhov, V. Matveev, V. Palichik, V. Perelygin, S. Shmatov, S. Shulha, N. Skatchkov, V. Smirnov, N. Voytishin, A. Zarubin, L. Chtchipounov, V. Golovtsov, Y. Ivanov, V. Kim, E. Kuznetsova, V. Murzin, V. Oreshkin, V. Sulimov, A. Vorobyev, Yu. Andreev, A. Dermenev, S. Gninenko, N. Golubev, A. Karneyeu, M. Kirsanov, N. Krasnikov, A. Pashenkov, D. Tlisov, A. Toropin, V. Epshteyn, V. Gavrilov, N. Lychkovskaya, V. Popov, I. Pozdnyakov, G. Safronov, A. Spiridonov, M. Toms, E. Vlasov, A. Zhokin, T. Aushev, A. Bylinkin, M. Chadeeva, R. Chistov, S. Polikarpov, V. Andreev, M. Azarkin, I. Dremin, M. Kirakosyan, A. Leonidov, A. Terkulov, A. Baskakov, A. Belyaev, E. Boos, A. Ershov, A. Gribushin, A. Kaminskiy, O. Kodolova, V. Korotkikh, I. Lokhtin, I. Miagkov, S. Obraztsov, S. Petrushanko, V. Savrin, A. Snigirev, I. Vardanyan, V. Blinov, Y. Skovpen, D. Shtol, I. Azhgirey, I. Bayshev, S. Bitioukov, D. Elumakhov, V. Kachanov, A. Kalinin, D. Konstantinov, V. Krychkine, V. Petrov, R. Ryutin, A. Sobol, S. Troshin, N. Tyurin, A. Uzunian, A. Volkov, P. Adzic, P. Cirkovic, D. Devetak, M. Dordevic, J. Milosevic, V. Rekovic, J. Alcaraz Maestre, M. Barrio Luna, E. Calvo, M. Cerrada, M. Chamizo Llatas, N. Colino, B. De La Cruz, A. Delgado Peris, A. Escalante Del Valle, C. Fernandez Bedoya, J. P. Fernández Ramos, J. Flix, M. C. Fouz, P. Garcia-Abia, O. Gonzalez Lopez, S. Goy Lopez, J. M. Hernandez, M. I. Josa, E. Navarro De Martino, A. Pérez-Calero Yzquierdo, J. Puerta Pelayo, A. Quintario Olmeda, I. Redondo, L. Romero, M. S. Soares, J. F. de Trocóniz, M. Missiroli, D. Moran, J. Cuevas, J. Fernandez Menendez, I. Gonzalez Caballero, J. R. González Fernández, E. Palencia Cortezon, S. Sanchez Cruz, I. Suárez Andrés, J. M. Vizan Garcia, I. J. Cabrillo, A. Calderon, E. Curras, M. Fernandez, J. Garcia-Ferrero, G. Gomez, A. Lopez Virto, J. Marco, C. Martinez Rivero, F. Matorras, J. Piedra Gomez, T. Rodrigo, A. Ruiz-Jimeno, L. Scodellaro, N. Trevisani, I. Vila, R. Vilar Cortabitarte, D. Abbaneo, E. Auffray, G. Auzinger, P. Baillon, A. H. Ball, D. Barney, P. Bloch, A. Bocci, C. Botta, T. Camporesi, R. Castello, M. Cepeda, G. Cerminara, Y. Chen, D. d’Enterria, A. Dabrowski, V. Daponte, A. David, M. De Gruttola, A. De Roeck, E. Di Marco, M. Dobson, B. Dorney, T. du Pree, D. Duggan, M. Dünser, N. Dupont, A. Elliott-Peisert, P. Everaerts, S. Fartoukh, G. Franzoni, J. Fulcher, W. Funk, D. Gigi, K. Gill, M. Girone, F. Glege, D. Gulhan, S. Gundacker, M. Guthoff, P. Harris, J. Hegeman, V. Innocente, P. Janot, J. Kieseler, H. Kirschenmann, V. Knünz, A. Kornmayer, M. J. Kortelainen, K. Kousouris, M. Krammer, C. Lange, P. Lecoq, C. Lourenço, M. T. Lucchini, L. Malgeri, M. Mannelli, A. Martelli, F. Meijers, J. A. Merlin, S. Mersi, E. Meschi, P. Milenovic, F. Moortgat, S. Morovic, M. Mulders, H. Neugebauer, S. Orfanelli, L. Orsini, L. Pape, E. Perez, M. Peruzzi, A. Petrilli, G. Petrucciani, A. Pfeiffer, M. Pierini, A. Racz, T. Reis, G. Rolandi, M. Rovere, H. Sakulin, J. B. Sauvan, C. Schäfer, C. Schwick, M. Seidel, A. Sharma, P. Silva, P. Sphicas, J. Steggemann, M. Stoye, Y. Takahashi, M. Tosi, D. Treille, A. Triossi, A. Tsirou, V. Veckalns, G. I. Veres, M. Verweij, N. Wardle, H. K. Wöhri, A. Zagozdzinska, W. D. Zeuner, W. Bertl, K. Deiters, W. Erdmann, R. Horisberger, Q. Ingram, H. C. Kaestli, D. Kotlinski, U. Langenegger, T. Rohe, S. A. Wiederkehr, F. Bachmair, L. Bäni, L. Bianchini, B. Casal, G. Dissertori, M. Dittmar, M. Donegà, C. Grab, C. Heidegger, D. Hits, J. Hoss, G. Kasieczka, W. Lustermann, B. Mangano, M. Marionneau, P. Martinez Ruiz del Arbol, M. Masciovecchio, M. T. Meinhard, D. Meister, F. Micheli, P. Musella, F. Nessi-Tedaldi, F. Pandolfi, J. Pata, F. Pauss, G. Perrin, L. Perrozzi, M. Quittnat, M. Rossini, M. Schönenberger, A. Starodumov, V. R. Tavolaro, K. Theofilatos, R. Wallny, T. K. Aarrestad, C. Amsler, L. Caminada, M. F. Canelli, A. De Cosa, C. Galloni, A. Hinzmann, T. Hreus, B. Kilminster, J. Ngadiuba, D. Pinna, G. Rauco, P. Robmann, D. Salerno, C. Seitz, Y. Yang, A. Zucchetta, V. Candelise, T. H. Doan, Sh. Jain, R. Khurana, M. Konyushikhin, C. M. Kuo, W. Lin, A. Pozdnyakov, S. S. Yu, Arun Kumar, P. Chang, Y. H. Chang, Y. Chao, K. F. Chen, P. H. Chen, F. Fiori, W.-S. Hou, Y. Hsiung, Y. F. Liu, R.-S. Lu, M. Miñano Moya, E. Paganis, A. Psallidas, J. F. Tsai, B. Asavapibhop, G. Singh, N. Srimanobhas, N. Suwonjandee, A. Adiguzel, S. Cerci, S. Damarseckin, Z. S. Demiroglu, C. Dozen, I. Dumanoglu, S. Girgis, G. Gokbulut, Y. Guler, I. Hos, E. E. Kangal, O. Kara, A. Kayis Topaksu, U. Kiminsu, M. Oglakci, G. Onengut, K. Ozdemir, D. Sunar Cerci, H. Topakli, S. Turkcapar, I. S. Zorbakir, C. Zorbilmez, B. Bilin, S. Bilmis, B. Isildak, G. Karapinar, M. Yalvac, M. Zeyrek, E. Gülmez, M. Kaya, O. Kaya, E. A. Yetkin, T. Yetkin, A. Cakir, K. Cankocak, S. Sen, B. Grynyov, L. Levchuk, P. Sorokin, R. Aggleton, F. Ball, L. Beck, J. J. Brooke, D. Burns, E. Clement, D. Cussans, H. Flacher, J. Goldstein, M. Grimes, G. P. Heath, H. F. Heath, J. Jacob, L. Kreczko, C. Lucas, D. M. Newbold, S. Paramesvaran, A. Poll, T. Sakuma, S. Seif El Nasr-storey, D. Smith, V. J. Smith, A. Belyaev, C. Brew, R. M. Brown, L. Calligaris, D. Cieri, D. J. A. Cockerill, J. A. Coughlan, K. Harder, S. Harper, E. Olaiya, D. Petyt, C. H. Shepherd-Themistocleous, A. Thea, I. R. Tomalin, T. Williams, M. Baber, R. Bainbridge, O. Buchmuller, A. Bundock, D. Burton, S. Casasso, M. Citron, D. Colling, L. Corpe, P. Dauncey, G. Davies, A. De Wit, M. Della Negra, R. Di Maria, P. Dunne, A. Elwood, D. Futyan, Y. Haddad, G. Hall, G. Iles, T. James, R. Lane, C. Laner, R. Lucas, L. Lyons, A.-M. Magnan, S. Malik, L. Mastrolorenzo, J. Nash, A. Nikitenko, J. Pela, B. Penning, M. Pesaresi, D. M. Raymond, A. Richards, A. Rose, E. Scott, C. Seez, S. Summers, A. Tapper, K. Uchida, M. Vazquez Acosta, T. Virdee, J. Wright, S. C. Zenz, J. E. Cole, P. R. Hobson, A. Khan, P. Kyberd, I. D. Reid, P. Symonds, L. Teodorescu, M. Turner, A. Borzou, K. Call, J. Dittmann, K. Hatakeyama, H. Liu, N. Pastika, R. Bartek, A. Dominguez, A. Buccilli, S. I. Cooper, C. Henderson, P. Rumerio, C. West, D. Arcaro, A. Avetisyan, T. Bose, D. Gastler, D. Rankin, C. Richardson, J. Rohlf, L. Sulak, D. Zou, G. Benelli, D. Cutts, A. Garabedian, J. Hakala, U. Heintz, J. M. Hogan, O. Jesus, K. H. M. Kwok, E. Laird, G. Landsberg, Z. Mao, M. Narain, S. Piperov, S. Sagir, E. Spencer, R. Syarif, R. Breedon, D. Burns, M. Calderon De La Barca Sanchez, S. Chauhan, M. Chertok, J. Conway, R. Conway, P. T. Cox, R. Erbacher, C. Flores, G. Funk, M. Gardner, W. Ko, R. Lander, C. Mclean, M. Mulhearn, D. Pellett, J. Pilot, S. Shalhout, M. Shi, J. Smith, M. Squires, D. Stolp, K. Tos, M. Tripathi, M. Bachtis, C. Bravo, R. Cousins, A. Dasgupta, A. Florent, J. Hauser, M. Ignatenko, N. Mccoll, D. Saltzberg, C. Schnaible, V. Valuev, M. Weber, E. Bouvier, K. Burt, R. Clare, J. Ellison, J. W. Gary, S. M. A. Ghiasi Shirazi, G. Hanson, J. Heilman, P. Jandir, E. Kennedy, F. Lacroix, O. R. Long, M. Olmedo Negrete, M. I. Paneva, A. Shrinivas, W. Si, H. Wei, S. Wimpenny, B. R. Yates, J. G. Branson, G. B. Cerati, S. Cittolin, M. Derdzinski, R. Gerosa, A. Holzner, D. Klein, V. Krutelyov, J. Letts, I. Macneill, D. Olivito, S. Padhi, M. Pieri, M. Sani, V. Sharma, S. Simon, M. Tadel, A. Vartak, S. Wasserbaech, C. Welke, J. Wood, F. Würthwein, A. Yagil, G. Zevi Della Porta, N. Amin, R. Bhandari, J. Bradmiller-Feld, C. Campagnari, A. Dishaw, V. Dutta, M. Franco Sevilla, C. George, F. Golf, L. Gouskos, J. Gran, R. Heller, J. Incandela, S. D. Mullin, A. Ovcharova, H. Qu, J. Richman, D. Stuart, I. Suarez, J. Yoo, D. Anderson, J. Bendavid, A. Bornheim, J. Bunn, J. Duarte, J. M. Lawhorn, A. Mott, H. B. Newman, C. Pena, M. Spiropulu, J. R. Vlimant, S. Xie, R. Y. Zhu, M. B. Andrews, T. Ferguson, M. Paulini, J. Russ, M. Sun, H. Vogel, I. Vorobiev, M. Weinberg, J. P. Cumalat, W. T. Ford, F. Jensen, A. Johnson, M. Krohn, S. Leontsinis, T. Mulholland, K. Stenson, S. R. Wagner, J. Alexander, J. Chaves, J. Chu, S. Dittmer, K. Mcdermott, N. Mirman, G. Nicolas Kaufman, J. R. Patterson, A. Rinkevicius, A. Ryd, L. Skinnari, L. Soffi, S. M. Tan, Z. Tao, J. Thom, J. Tucker, P. Wittich, M. Zientek, D. Winn, S. Abdullin, M. Albrow, G. Apollinari, A. Apresyan, S. Banerjee, L. A. T. Bauerdick, A. Beretvas, J. Berryhill, P. C. Bhat, G. Bolla, K. Burkett, J. N. Butler, H. W. K. Cheung, F. Chlebana, S. Cihangir, M. Cremonesi, V. D. Elvira, I. Fisk, J. Freeman, E. Gottschalk, L. Gray, D. Green, S. Grünendahl, O. Gutsche, D. Hare, R. M. Harris, S. Hasegawa, J. Hirschauer, Z. Hu, B. Jayatilaka, S. Jindariani, M. Johnson, U. Joshi, B. Klima, B. Kreis, S. Lammel, J. Linacre, D. Lincoln, R. Lipton, M. Liu, T. Liu, R. Lopes De Sá, J. Lykken, K. Maeshima, N. Magini, J. M. Marraffino, S. Maruyama, D. Mason, P. McBride, P. Merkel, S. Mrenna, S. Nahn, V. O’Dell, K. Pedro, O. Prokofyev, G. Rakness, L. Ristori, E. Sexton-Kennedy, A. Soha, W. J. Spalding, L. Spiegel, S. Stoynev, J. Strait, N. Strobbe, L. Taylor, S. Tkaczyk, N. V. Tran, L. Uplegger, E. W. Vaandering, C. Vernieri, M. Verzocchi, R. Vidal, M. Wang, H. A. Weber, A. Whitbeck, Y. Wu, D. Acosta, P. Avery, P. Bortignon, D. Bourilkov, A. Brinkerhoff, A. Carnes, M. Carver, D. Curry, S. Das, R. D. Field, I. K. Furic, J. Konigsberg, A. Korytov, J. F. Low, P. Ma, K. Matchev, H. Mei, G. Mitselmakher, D. Rank, L. Shchutska, D. Sperka, L. Thomas, J. Wang, S. Wang, J. Yelton, S. Linn, P. Markowitz, G. Martinez, J. L. Rodriguez, A. Ackert, T. Adams, A. Askew, S. Bein, S. Hagopian, V. Hagopian, K. F. Johnson, H. Prosper, A. Santra, R. Yohay, M. M. Baarmand, V. Bhopatkar, S. Colafranceschi, M. Hohlmann, D. Noonan, T. Roy, F. Yumiceva, M. R. Adams, L. Apanasevich, D. Berry, R. R. Betts, I. Bucinskaite, R. Cavanaugh, O. Evdokimov, L. Gauthier, C. E. Gerber, D. J. Hofman, K. Jung, I. D. Sandoval Gonzalez, N. Varelas, H. Wang, Z. Wu, M. Zakaria, J. Zhang, B. Bilki, W. Clarida, K. Dilsiz, S. Durgut, R. P. Gandrajula, M. Haytmyradov, V. Khristenko, J.-P. Merlo, H. Mermerkaya, A. Mestvirishvili, A. Moeller, J. Nachtman, H. Ogul, Y. Onel, F. Ozok, A. Penzo, C. Snyder, E. Tiras, J. Wetzel, K. Yi, I. Anderson, B. Blumenfeld, A. Cocoros, N. Eminizer, D. Fehling, L. Feng, A. V. Gritsan, P. Maksimovic, J. Roskes, U. Sarica, M. Swartz, M. Xiao, Y. Xin, C. You, A. Al-bataineh, P. Baringer, A. Bean, S. Boren, J. Bowen, J. Castle, L. Forthomme, R. P. Kenny, S. Khalil, A. Kropivnitskaya, D. Majumder, W. Mcbrayer, M. Murray, S. Sanders, R. Stringer, J. D. Tapia Takaki, Q. Wang, A. Ivanov, K. Kaadze, Y. Maravin, A. Mohammadi, L. K. Saini, N. Skhirtladze, S. Toda, F. Rebassoo, D. Wright, C. Anelli, A. Baden, O. Baron, A. Belloni, B. Calvert, S. C. Eno, C. Ferraioli, J. A. Gomez, N. J. Hadley, S. Jabeen, G. Y. Jeng, R. G. Kellogg, T. Kolberg, J. Kunkle, A. C. Mignerey, F. Ricci-Tam, Y. H. Shin, A. Skuja, M. B. Tonjes, S. C. Tonwar, D. Abercrombie, B. Allen, A. Apyan, V. Azzolini, R. Barbieri, A. Baty, R. Bi, K. Bierwagen, S. Brandt, W. Busza, I. A. Cali, M. D’Alfonso, Z. Demiragli, L. Di Matteo, G. Gomez Ceballos, M. Goncharov, D. Hsu, Y. Iiyama, G. M. Innocenti, M. Klute, D. Kovalskyi, K. Krajczar, Y. S. Lai, Y.-J. Lee, A. Levin, P. D. Luckey, B. Maier, A. C. Marini, C. Mcginn, C. Mironov, S. Narayanan, X. Niu, C. Paus, C. Roland, G. Roland, J. Salfeld-Nebgen, G. S. F. Stephans, K. Tatar, M. Varma, D. Velicanu, J. Veverka, J. Wang, T. W. Wang, B. Wyslouch, M. Yang, A. C. Benvenuti, R. M. Chatterjee, A. Evans, P. Hansen, S. Kalafut, S. C. Kao, Y. Kubota, Z. Lesko, J. Mans, S. Nourbakhsh, N. Ruckstuhl, R. Rusack, N. Tambe, J. Turkewitz, J. G. Acosta, S. Oliveros, E. Avdeeva, K. Bloom, D. R. Claes, C. Fangmeier, R. Gonzalez Suarez, R. Kamalieddin, I. Kravchenko, A. Malta Rodrigues, J. Monroy, J. E. Siado, G. R. Snow, B. Stieger, M. Alyari, J. Dolen, A. Godshalk, C. Harrington, I. Iashvili, J. Kaisen, D. Nguyen, A. Parker, S. Rappoccio, B. Roozbahani, G. Alverson, E. Barberis, A. Hortiangtham, A. Massironi, D. M. Morse, D. Nash, T. Orimoto, R. Teixeira De Lima, D. Trocino, R.-J. Wang, D. Wood, S. Bhattacharya, O. Charaf, K. A. Hahn, A. Kumar, N. Mucia, N. Odell, B. Pollack, M. H. Schmitt, K. Sung, M. Trovato, M. Velasco, N. Dev, M. Hildreth, K. Hurtado Anampa, C. Jessop, D. J. Karmgard, N. Kellams, K. Lannon, N. Marinelli, F. Meng, C. Mueller, Y. Musienko, M. Planer, A. Reinsvold, R. Ruchti, N. Rupprecht, G. Smith, S. Taroni, M. Wayne, M. Wolf, A. Woodard, J. Alimena, L. Antonelli, B. Bylsma, L. S. Durkin, S. Flowers, B. Francis, A. Hart, C. Hill, R. Hughes, W. Ji, B. Liu, W. Luo, D. Puigh, B. L. Winer, H. W. Wulsin, S. Cooperstein, O. Driga, P. Elmer, J. Hardenbrook, P. Hebda, D. Lange, J. Luo, D. Marlow, T. Medvedeva, K. Mei, I. Ojalvo, J. Olsen, C. Palmer, P. Piroué, D. Stickland, A. Svyatkovskiy, C. Tully, S. Malik, A. Barker, V. E. Barnes, S. Folgueras, L. Gutay, M. K. Jha, M. Jones, A. W. Jung, A. Khatiwada, D. H. Miller, N. Neumeister, J. F. Schulte, X. Shi, J. Sun, F. Wang, W. Xie, N. Parashar, J. Stupak, A. Adair, B. Akgun, Z. Chen, K. M. Ecklund, F. J. M. Geurts, M. Guilbaud, W. Li, B. Michlin, M. Northup, B. P. Padley, J. Roberts, J. Rorie, Z. Tu, J. Zabel, B. Betchart, A. Bodek, P. de Barbaro, R. Demina, Y. T. Duh, T. Ferbel, M. Galanti, A. Garcia-Bellido, J. Han, O. Hindrichs, A. Khukhunaishvili, K. H. Lo, P. Tan, M. Verzetti, A. Agapitos, J. P. Chou, Y. Gershtein, T. A. Gómez Espinosa, E. Halkiadakis, M. Heindl, E. Hughes, S. Kaplan, R. Kunnawalkam Elayavalli, S. Kyriacou, A. Lath, K. Nash, M. Osherson, H. Saka, S. Salur, S. Schnetzer, D. Sheffield, S. Somalwar, R. Stone, S. Thomas, P. Thomassen, M. Walker, A. G. Delannoy, M. Foerster, J. Heideman, G. Riley, K. Rose, S. Spanier, K. Thapa, O. Bouhali, A. Celik, M. Dalchenko, M. De Mattia, A. Delgado, S. Dildick, R. Eusebi, J. Gilmore, T. Huang, E. Juska, T. Kamon, R. Mueller, Y. Pakhotin, R. Patel, A. Perloff, L. Perniè, D. Rathjens, A. Safonov, A. Tatarinov, K. A. Ulmer, N. Akchurin, C. Cowden, J. Damgov, F. De Guio, C. Dragoiu, P. R. Dudero, J. Faulkner, E. Gurpinar, S. Kunori, K. Lamichhane, S. W. Lee, T. Libeiro, T. Peltola, S. Undleeb, I. Volobouev, Z. Wang, S. Greene, A. Gurrola, R. Janjam, W. Johns, C. Maguire, A. Melo, H. Ni, P. Sheldon, S. Tuo, J. Velkovska, Q. Xu, M. W. Arenton, P. Barria, B. Cox, J. Goodell, R. Hirosky, A. Ledovskoy, H. Li, C. Neu, T. Sinthuprasith, X. Sun, Y. Wang, E. Wolfe, F. Xia, C. Clarke, R. Harr, P. E. Karchin, J. Sturdy, D. A. Belknap, J. Buchanan, C. Caillol, S. Dasu, L. Dodd, S. Duric, B. Gomber, M. Grothe, M. Herndon, A. Hervé, P. Klabbers, A. Lanaro, A. Levine, K. Long, R. Loveless, T. Perry, G. A. Pierro, G. Polese, T. Ruggles, A. Savin, N. Smith, W. H. Smith, D. Taylor, N. Woods, CMS Collaboration

**Affiliations:** 1 Yerevan Physics Institute, Yerevan, Armenia; 2 Institut für Hochenergiephysik, Vienna, Austria; 3 Institute for Nuclear Problems, Minsk, Belarus; 4 National Centre for Particle and High Energy Physics, Minsk, Belarus; 5 Universiteit Antwerpen, Antwerpen, Belgium; 6 Vrije Universiteit Brussel, Brussel, Belgium; 7 Université Libre de Bruxelles, Bruxelles, Belgium; 8 Ghent University, Ghent, Belgium; 9 Université Catholique de Louvain, Louvain-la-Neuve, Belgium; 10 Université de Mons, Mons, Belgium; 11 Centro Brasileiro de Pesquisas Fisicas, Rio de Janeiro, Brazil; 12 Universidade do Estado do Rio de Janeiro, Rio de Janeiro, Brazil; 13 Universidade Estadual Paulista, Universidade Federal do ABC, São Paulo, Brazil; 14 Institute for Nuclear Research and Nuclear Energy, Sofia, Bulgaria; 15 University of Sofia, Sofia, Bulgaria; 16 Beihang University, Beijing, China; 17 Institute of High Energy Physics, Beijing, China; 18 State Key Laboratory of Nuclear Physics and Technology, Peking University, Beijing, China; 19 Universidad de Los Andes, Bogotá, Colombia; 20 Faculty of Electrical Engineering, Mechanical Engineering and Naval Architecture, University of Split, Split, Croatia; 21 Faculty of Science, University of Split, Split, Croatia; 22 Institute Rudjer Boskovic, Zagreb, Croatia; 23 University of Cyprus, Nicosia, Cyprus; 24 Charles University, Prague, Czech Republic; 25 Universidad San Francisco de Quito, Quito, Ecuador; 26 Academy of Scientific Research and Technology of the Arab Republic of Egypt, Egyptian Network of High Energy Physics, Cairo, Egypt; 27 National Institute of Chemical Physics and Biophysics, Tallinn, Estonia; 28 Department of Physics, University of Helsinki, Helsinki, Finland; 29 Helsinki Institute of Physics, Helsinki, Finland; 30 Lappeenranta University of Technology, Lappeenranta, Finland; 31 IRFU, CEA, Université Paris-Saclay, Gif-sur-Yvette, France; 32 Laboratoire Leprince-Ringuet, Ecole Polytechnique, IN2P3-CNRS, Palaiseau, France; 33 Institut Pluridisciplinaire Hubert Curien (IPHC), Université de Strasbourg, CNRS-IN2P3, Strasbourg, France; 34 Centre de Calcul de l’Institut National de Physique Nucleaire et de Physique des Particules, CNRS/IN2P3, Villeurbanne, France; 35 Université de Lyon, Université Claude Bernard Lyon 1, CNRS-IN2P3, Institut de Physique Nucléaire de Lyon, Villeurbanne, France; 36 Georgian Technical University, Tbilisi, Georgia; 37 Tbilisi State University, Tbilisi, Georgia; 38 RWTH Aachen University, I. Physikalisches Institut, Aachen, Germany; 39 RWTH Aachen University, III. Physikalisches Institut A, Aachen, Germany; 40 RWTH Aachen University, III. Physikalisches Institut B, Aachen, Germany; 41 Deutsches Elektronen-Synchrotron, Hamburg, Germany; 42 University of Hamburg, Hamburg, Germany; 43 Institut für Experimentelle Kernphysik, Karlsruhe, Germany; 44 Institute of Nuclear and Particle Physics (INPP), NCSR Demokritos, Aghia Paraskevi, Greece; 45 National and Kapodistrian University of Athens, Athens, Greece; 46 University of Ioánnina, Ioannina, Greece; 47 MTA-ELTE Lendület CMS Particle and Nuclear Physics Group, Eötvös Loránd University, Budapest, Hungary; 48 Wigner Research Centre for Physics, Budapest, Hungary; 49 Institute of Nuclear Research ATOMKI, Debrecen, Hungary; 50 Institute of Physics, University of Debrecen, Debrecen, Hungary; 51 Indian Institute of Science (IISc), Bangalore, India; 52 National Institute of Science Education and Research, Bhubaneswar, India; 53 Panjab University, Chandigarh, India; 54 University of Delhi, Delhi, India; 55 Saha Institute of Nuclear Physics, Kolkata, India; 56 Indian Institute of Technology Madras, Madras, India; 57 Bhabha Atomic Research Centre, Mumbai, India; 58 Tata Institute of Fundamental Research-A, Mumbai, India; 59 Tata Institute of Fundamental Research-B, Mumbai, India; 60 Indian Institute of Science Education and Research (IISER), Pune, India; 61 Institute for Research in Fundamental Sciences (IPM), Tehran, Iran; 62 University College Dublin, Dublin, Ireland; 63 INFN Sezione di Bari, Università di Bari, Politecnico di Bari, Bari, Italy; 64 INFN Sezione di Bologna, Università di Bologna, Bologna, Italy; 65 INFN Sezione di Catania, Università di Catania, Catania, Italy; 66 INFN Sezione di Firenze, Università di Firenze, Florence, Italy; 67 INFN Laboratori Nazionali di Frascati, Frascati, Italy; 68 INFN Sezione di Genova, Università di Genova, Genoa, Italy; 69 INFN Sezione di Milano-Bicocca, Università di Milano-Bicocca, Milan, Italy; 70 INFN Sezione di Napoli, Università di Napoli ’Federico II’, Napoli, Italy, Università della Basilicata, Potenza, Italy, Università G. Marconi, Rome, Italy; 71 INFN Sezione di Padova, Università di Padova, Padova, Italy, Università di Trento, Trento, Italy; 72 INFN Sezione di Pavia, Università di Pavia, Pavia, Italy; 73 INFN Sezione di Perugia, Università di Perugia, Perugia, Italy; 74 INFN Sezione di Pisa, Università di Pisa, Scuola Normale Superiore di Pisa, Pisa, Italy; 75 INFN Sezione di Roma, Università di Roma, Rome, Italy; 76 INFN Sezione di Torino, Università di Torino, Torino, Italy, Università del Piemonte Orientale, Novara, Italy; 77 INFN Sezione di Trieste, Università di Trieste, Trieste, Italy; 78 Kyungpook National University, Taegu, Korea; 79 Chonbuk National University, Jeonju, Korea; 80 Institute for Universe and Elementary Particles, Chonnam National University, Kwangju, Korea; 81 Hanyang University, Seoul, Korea; 82 Korea University, Seoul, Korea; 83 Seoul National University, Seoul, Korea; 84 University of Seoul, Seoul, Korea; 85 Sungkyunkwan University, Suwon, Korea; 86 Vilnius University, Vilnius, Lithuania; 87 National Centre for Particle Physics, Universiti Malaya, Kuala Lumpur, Malaysia; 88 Centro de Investigacion y de Estudios Avanzados del IPN, Mexico City, Mexico; 89 Universidad Iberoamericana, Mexico City, Mexico; 90 Benemerita Universidad Autonoma de Puebla, Puebla, Mexico; 91 Universidad Autónoma de San Luis Potosí, San Luis Potosí, Mexico; 92 University of Auckland, Auckland, New Zealand; 93 University of Canterbury, Christchurch, New Zealand; 94 National Centre for Physics, Quaid-I-Azam University, Islamabad, Pakistan; 95 National Centre for Nuclear Research, Swierk, Poland; 96 Institute of Experimental Physics, Faculty of Physics, University of Warsaw, Warsaw, Poland; 97 Laboratório de Instrumentação e Física Experimental de Partículas, Lisbon, Portugal; 98 Joint Institute for Nuclear Research, Dubna, Russia; 99 Petersburg Nuclear Physics Institute, Gatchina (St. Petersburg), Russia; 100 Institute for Nuclear Research, Moscow, Russia; 101 Institute for Theoretical and Experimental Physics, Moscow, Russia; 102 Moscow Institute of Physics and Technology, Moscow, Russia; 103 National Research Nuclear University ‘Moscow Engineering Physics Institute’ (MEPhI), Moscow, Russia; 104 P.N. Lebedev Physical Institute, Moscow, Russia; 105 Skobeltsyn Institute of Nuclear Physics, Lomonosov Moscow State University, Moscow, Russia; 106 Novosibirsk State University (NSU), Novosibirsk, Russia; 107 State Research Center of Russian Federation, Institute for High Energy Physics, Protvino, Russia; 108 University of Belgrade, Faculty of Physics and Vinca Institute of Nuclear Sciences, Belgrade, Serbia; 109 Centro de Investigaciones Energéticas Medioambientales y Tecnológicas (CIEMAT), Madrid, Spain; 110 Universidad Autónoma de Madrid, Madrid, Spain; 111 Universidad de Oviedo, Oviedo, Spain; 112 Instituto de Física de Cantabria (IFCA), CSIC-Universidad de Cantabria, Santander, Spain; 113 CERN, European Organization for Nuclear Research, Geneva, Switzerland; 114 Paul Scherrer Institut, Villigen, Switzerland; 115 Institute for Particle Physics, ETH Zurich, Zurich, Switzerland; 116 Universität Zürich, Zurich, Switzerland; 117 National Central University, Chung-Li, Taiwan; 118 National Taiwan University (NTU), Taipei, Taiwan; 119 Department of Physics, Faculty of Science, Chulalongkorn University, Bangkok, Thailand; 120 Physics Department, Science and Art Faculty, Cukurova University, Adana, Turkey; 121 Physics Department, Middle East Technical University, Ankara, Turkey; 122 Bogazici University, Istanbul, Turkey; 123 Istanbul Technical University, Istanbul, Turkey; 124 Institute for Scintillation Materials of National Academy of Science of Ukraine, Kharkov, Ukraine; 125 National Scientific Center, Kharkov Institute of Physics and Technology, Kharkov, Ukraine; 126 University of Bristol, Bristol, UK; 127 Rutherford Appleton Laboratory, Didcot, UK; 128 Imperial College, London, UK; 129 Brunel University, Uxbridge, UK; 130 Baylor University, Waco, USA; 131 Catholic University of America, Washington, D.C., USA; 132 The University of Alabama, Tuscaloosa, USA; 133 Boston University, Boston, USA; 134 Brown University, Providence, USA; 135 University of California, Davis, Davis, USA; 136 University of California, Los Angeles, USA; 137 University of California, Riverside, Riverside, USA; 138 University of California, San Diego, La Jolla, USA; 139 Department of Physics, University of California, Santa Barbara, Santa Barbara, USA; 140 California Institute of Technology, Pasadena, USA; 141 Carnegie Mellon University, Pittsburgh, USA; 142 University of Colorado Boulder, Boulder, USA; 143 Cornell University, Ithaca, USA; 144 Fairfield University, Fairfield, USA; 145 Fermi National Accelerator Laboratory, Batavia, USA; 146 University of Florida, Gainesville, USA; 147 Florida International University, Miami, USA; 148 Florida State University, Tallahassee, USA; 149 Florida Institute of Technology, Melbourne, USA; 150 University of Illinois at Chicago (UIC), Chicago, USA; 151 The University of Iowa, Iowa City, USA; 152 Johns Hopkins University, Baltimore, USA; 153 The University of Kansas, Lawrence, USA; 154 Kansas State University, Manhattan, USA; 155 Lawrence Livermore National Laboratory, Livermore, USA; 156 University of Maryland, College Park, USA; 157 Massachusetts Institute of Technology, Cambridge, USA; 158 University of Minnesota, Minneapolis, USA; 159 University of Mississippi, Oxford, USA; 160 University of Nebraska-Lincoln, Lincoln, USA; 161 State University of New York at Buffalo, Buffalo, USA; 162 Northeastern University, Boston, USA; 163 Northwestern University, Evanston, USA; 164 University of Notre Dame, Notre Dame, USA; 165 The Ohio State University, Columbus, USA; 166 Princeton University, Princeton, USA; 167 University of Puerto Rico, Mayagüez, USA; 168 Purdue University, West Lafayette, USA; 169 Purdue University Calumet, Hammond, USA; 170 Rice University, Houston, USA; 171 University of Rochester, Rochester, USA; 172 Rutgers, The State University of New Jersey, Piscataway, USA; 173 University of Tennessee, Knoxville, USA; 174 Texas A&M University, College Station, USA; 175 Texas Tech University, Lubbock, USA; 176 Vanderbilt University, Nashville, USA; 177 University of Virginia, Charlottesville, USA; 178 Wayne State University, Detroit, USA; 179 University of Wisconsin-Madison, Madison, WI USA; 180 CERN, 1211 Geneva 23, Switzerland

## Abstract

This paper reports the measurement of $\mathrm{J}/{\psi }$ meson production in proton–proton ($\mathrm {p}\mathrm {p}$) and proton–lead ($\mathrm {p}\mathrm {Pb}$) collisions at a center-of-mass energy per nucleon pair of $5.02\,\text {TeV}$ by the CMS experiment at the LHC. The data samples used in the analysis correspond to integrated luminosities of 28$\,\text {pb}^{-1}$ and 35$\,\text {nb}^{-1}$ for $\mathrm {p}\mathrm {p}$ and $\mathrm {p}\mathrm {Pb}$ collisions, respectively. Prompt and nonprompt $\mathrm{J}/{\psi }$ mesons, the latter produced in the decay of ${\mathrm {B}}$ hadrons, are measured in their dimuon decay channels. Differential cross sections are measured in the transverse momentum range of $2<p_{\mathrm {T}} <30{\,\text {GeV}/{c}}$, and center-of-mass rapidity ranges of $|y_\mathrm{{CM}} |<2.4$ ($\mathrm {p}\mathrm {p}$) and $-2.87<y_\mathrm{{CM}}<1.93$ ($\mathrm {p}\mathrm {Pb}$). The nuclear modification factor, $R_{\mathrm {p}\mathrm {Pb}}$, is measured as a function of both $p_{\mathrm {T}}$ and $y_\mathrm{{CM}}$. Small modifications to the $\mathrm{J}/{\psi }$ cross sections are observed in $\mathrm {p}\mathrm {Pb}$ relative to $\mathrm {p}\mathrm {p}$ collisions. The ratio of $\mathrm{J}/{\psi }$ production cross sections in $\mathrm {p}$-going and Pb-going directions, $R_\mathrm{{FB}}$, studied as functions of $p_{\mathrm {T}}$ and $y_\mathrm{{CM}}$, shows a significant decrease for increasing transverse energy deposited at large pseudorapidities. These results, which cover a wide kinematic range, provide new insight on the role of cold nuclear matter effects on prompt and nonprompt $\mathrm{J}/{\psi }$ production.

## Introduction

It was suggested 3 decades ago that quark-gluon plasma (QGP) formation would suppress the yield of $\mathrm{J}/{\psi }$ mesons in high-energy heavy ion collisions, relative to that in proton–proton ($\mathrm {p}\mathrm {p}$) collisions, as a consequence of Debye screening of the heavy-quark potential at finite temperature [1]. This QGP signature triggered intense research activity, both experimental and theoretical, on the topic of heavy quarkonium production in nuclear collisions. Experiments at SPS [2, 3], RHIC [4, 5], and the CERN LHC [6, 7] have reported a significant $\mathrm{J}/{\psi }$ suppression in heavy ion collisions compared to the expectation based on $\mathrm {p}\mathrm {p}$ data. This suppression is found to be larger for more central collisions over a wide range in rapidity (*y*) and transverse momentum ($p_{\mathrm {T}}$). In addition, a suppression of different bottomonium states $[{\varUpsilon \mathrm{(1S)}},\,{\varUpsilon \mathrm{(2S)}},\,{\varUpsilon \mathrm{(3S)}}]$ has been observed at the LHC in lead–lead ($\mathrm {PbPb}$) collisions at a center-of-mass energy per nucleon pair of $\sqrt{s_{\mathrm {NN}}} =2.76\,\text {TeV}$ [8–10], which appears to be consistent with the suggested picture of quarkonium suppression in the QGP [11, 12].

In order to interpret these results unambiguously, it is necessary to constrain the so-called cold nuclear matter effects on quarkonium production, through, e.g., baseline measurements in $\mathrm {p}\mathrm {Pb}$ collisions. Among these effects, parton distribution functions in nuclei (nPDF) are known to differ from those in a free proton and thus influence the quarkonium yields in nuclear collisions. The expected depletion of nuclear gluon density at small values of the momentum fraction (*x*), an effect known as shadowing, would suppress $\mathrm{J}/{\psi }$ production at forward *y*, corresponding to the $\mathrm {p}$-going direction in $\mathrm {p}\mathrm {Pb}$ collisions [13, 14]. It has been also suggested that gluon radiation induced by parton multiple scattering in the nucleus can lead to $p_{\mathrm {T}}$ broadening and coherent energy loss, resulting in a significant forward $\mathrm{J}/{\psi }$ suppression in $\mathrm {p}\mathrm {Pb}$ collisions at all available energies [15, 16]. These phenomena can be quantified by the nuclear modification factor, $R_{\mathrm {p}\mathrm {Pb}}$, defined as the ratio of $\mathrm{J}/{\psi }$ cross sections in $\mathrm {p}\mathrm {Pb}$ collisions over those in $\mathrm {p}\mathrm {p}$ collisions scaled by the number of nucleons in the $\mathrm {Pb}$ ion ($\mathrm {A}=208$), and by the $R_{\mathrm {FB}}$ ratio of $\mathrm{J}/{\psi }$ cross sections at forward ($\mathrm {p}$-going direction) over those at backward ($\mathrm {Pb}$-going direction) rapidities.

In addition to prompt $\mathrm{J}/{\psi }$ mesons, directly produced in the primary interaction or from the decay of heavier charmonium states such as $\mathrm {\psi (2S)}$ and $\chi _\text {c}$, the production of $\mathrm{J}/{\psi }$ mesons includes a nonprompt contribution coming from the later decay of ${\mathrm {B}}$ hadrons, whose production rates are also expected to be affected by cold nuclear matter effects [17, 18]. However, neither high-$p_{\mathrm {T}}$ ${\mathrm {B}}$ mesons nor b quark jets show clear evidence of their cross sections being modified in $\mathrm {p}\mathrm {Pb}$ collisions [19, 20]. In this respect, the nonprompt component of $\mathrm{J}/{\psi }$ production can shed light on the nature of nuclear effects (if any) on bottom-quark production at low $p_{\mathrm {T}}$.

At the LHC, $\mathrm{J}/{\psi }$ meson production in $\mathrm {p}\mathrm {Pb}$ collisions at $\sqrt{s_{\mathrm {NN}}} =5.02\,\text {TeV}$ has been measured by the ALICE [21, 22], ATLAS [23], and LHCb [24] collaborations. The $R_{\mathrm {FB}}$ ratio has been determined as functions of rapidity in the center-of-mass frame, $y_{\mathrm {CM}}$, and $p_{\mathrm {T}}$. Using an interpolation of the $\mathrm {p}\mathrm {p}$ production cross sections at the same collision energy, $R_{\mathrm {p}\mathrm {Pb}}$ has also been estimated in Refs. [21, 22, 24] as functions of $y_{\mathrm {CM}}$ and $p_{\mathrm {T}}$. A significant suppression of the prompt $\mathrm{J}/{\psi }$ production in $\mathrm {p}\mathrm {Pb}$ collisions has been observed at forward $y_{\mathrm {CM}}$ and low $p_{\mathrm {T}}$, while no strong nuclear effects are observed at backward $y_{\mathrm {CM}}$.

This paper reports an analysis of $\mathrm{J}/{\psi }$ production in $\mathrm {p}\mathrm {p}$ and $\mathrm {p}\mathrm {Pb}$ collisions at $\sqrt{s_{\mathrm {NN}}} =5.02\,\text {TeV}$, using data collected with the CMS detector in 2013 ($\mathrm {p}\mathrm {Pb}$) and in 2015 ($\mathrm {p}\mathrm {p}$). The $\mathrm{J}/{\psi }$ mesons with $2<p_{\mathrm {T}} <30{\,\text {GeV}/{c}}$ are measured via their dimuon decay channels in ranges of $|y_{\mathrm {CM}} |<2.4$ in $\mathrm {p}\mathrm {p}$ and $-2.87<y_{\mathrm {CM}} <1.93$ in $\mathrm {p}\mathrm {Pb}$ collisions. The corresponding values of *x* range from $10^{-4}$, at forward $y_{\mathrm {CM}}$ and low $p_{\mathrm {T}}$, to $10^{-2}$, at backward $y_{\mathrm {CM}}$ and higher $p_{\mathrm {T}}$. Both $R_{\mathrm {p}\mathrm {Pb}}$ and $R_{\mathrm {FB}}$ are measured as functions of $y_{\mathrm {CM}}$ and $p_{\mathrm {T}}$. The latter ratio is also studied as a function of the event activity in $\mathrm {p}\mathrm {Pb}$ collisions, as characterized by the transverse energy deposited in the CMS detector at large pseudorapidities.

## Experimental setup and event selection

The main feature of the CMS detector is a superconducting solenoid with an internal diameter of 6 m, providing a magnetic field of 3.8 T. Within the field volume are the silicon pixel and strip tracker, the crystal electromagnetic calorimeter, and the brass and scintillator hadronic calorimeter. The silicon pixel and strip tracker measures charged particle trajectories in the pseudorapidity range of $|\eta |<2.5$. It consists of 66 M pixel and 10 M strip sensor elements. Muons are detected in the range of $|\eta |<2.4$, with detection planes based on three technologies: drift tubes, cathode strip chambers, and resistive plate chambers. The CMS apparatus also has extensive forward calorimetry, including two steel and quartz-fiber Cherenkov hadron forward (HF) calorimeters, which cover $2.9<|\eta |<5.2$. These detectors are used for online event selection and the impact parameter characterization of the events in $\mathrm {p}\mathrm {Pb}$ collisions, where the term impact parameter refers to the transverse distance between the two centers of the colliding hadrons. A more detailed description of the CMS detector, together with a definition of the coordinate system used and the relevant kinematic variables, can be found in Ref. [25].

The $\mathrm {p}\mathrm {Pb}$ data set used in this analysis corresponds to an integrated luminosity of 34.6$\,\text {nb}^{-1}$. The beam energies are 4$\,\text {TeV}$ for $\mathrm {p}$, and 1.58$\,\text {TeV}$ per nucleon for the $\mathrm {Pb}$ nuclei, resulting in $\sqrt{s_{\mathrm {NN}}} =5.02\,\text {TeV}$. The direction of the higher-energy $\mathrm {p}$ beam was initially set up to be clockwise, and was reversed after 20.7$\,\text {nb}^{-1}$. As a result of the beam energy difference, the nucleon–nucleon center-of-mass in $\mathrm {p}\mathrm {Pb}$ collisions is not at rest with respect to the laboratory frame. Massless particles emitted at $|\eta _{\mathrm {CM}} |=0$ in the nucleon–nucleon center-of-mass frame are detected at $\eta _{\text {lab}}=-0.465$ for the first run period (clockwise $\mathrm {p}$ beam) and $+0.465$ for the second run period (counterclockwise $\mathrm {p}$ beam) in the laboratory frame; the region $-2.87<y_{\mathrm {CM}} <1.93$ is thus probed by flipping the $\eta$ of one data set so that the $\mathrm {p}$-going direction is always toward positive $y_{\mathrm {CM}}$. The $\mathrm {p}\mathrm {p}$ data set is also collected at the same collision energy with an integrated luminosity of 28.0$\,\text {pb}^{-1}$. In this sample, $\mathrm{J}/{\psi }$ mesons are measured over $|y_{\mathrm {CM}} |<2.4$.

In order to remove beam-related background such as beam-gas interactions, inelastic hadronic collisions are selected by requiring a coincidence of at least one of the HF calorimeter towers with more than 3$\,\text {GeV}$ of total energy on each side of the interaction point. This requirement is not present in $\mathrm {p}\mathrm {p}$ collisions which suffer less from photon-induced interactions compared to $\mathrm {p}\mathrm {Pb}$ collisions. The $\mathrm {p}\mathrm {p}$ and $\mathrm {p}\mathrm {Pb}$ events are further selected to have at least one reconstructed primary vertex composed of two or more associated tracks, excluding the two muons from the $\mathrm{J}/{\psi }$ candidates, within 25$\,\text {cm}$ from the nominal interaction point along the beam axis and within 2$\,\text {cm}$ in its transverse plane. To reject beam-scraping events, the fraction of good-quality tracks associated with the primary vertex is required to be larger than 25% when there are more than 10 tracks per event.

In $\mathrm {p}\mathrm {Pb}$ collisions, an additional filter [26] is applied to remove events containing multiple interactions per bunch crossing (pileup). After the selection, the residual fraction of pileup events is reduced from 3% to less than 0.2%. This pileup rejection results in a 4.1% signal loss, which is corrected for in the cross section measurements. Since pileup only affects the event activity dependence in $\mathrm {p}\mathrm {Pb}$ results, no filter is applied in $\mathrm {p}\mathrm {p}$ results.

Dimuon events are selected by the level-1 trigger, a hardware-based trigger system requiring two muon candidates in the muon detectors with no explicit limitations in $p_{\mathrm {T}}$ or *y*. In the offline analysis, muons are required to be within the following kinematic regions, which ensure single-muon reconstruction efficiencies above 10%:

1 $$\begin{aligned} \begin{array}{lll} &{}p_{\mathrm {T}} ^{\mu }>3.3{\,\text {GeV}/{c}} &{} \text { for }|\eta _{\text {lab}}^{\mu } |<1.2,\\ &{}p_{\mathrm {T}} ^{\mu }>(4.0-1.1|\eta _{\text {lab}}^{\mu } |){\,\text {GeV}/{c}} &{} \text { for }1.2\le |\eta _{\text {lab}}^{\mu } |<2.1,\\ &{}p_{\mathrm {T}} ^{\mu }>1.3{\,\text {GeV}/{c}} &{}\text { for }2.1\le |\eta _{\text {lab}}^{\mu } |<2.4.\\ \end{array} \end{aligned}$$

The muon pairs are further selected to be of opposite charge, to originate from a common vertex with a $\chi ^2$ probability greater than 1%, and to match standard identification criteria [27].

Simulated events are used to obtain the correction factors for acceptance and efficiency. The Monte Carlo (MC) samples of $\mathrm{J}/{\psi }$ mesons are generated using pythia 8.209 [28] for $\mathrm {p}\mathrm {p}$ and pythia 6.424 [29] for $\mathrm {p}\mathrm {Pb}$ collisions. Generated particles in the $\mathrm {p}\mathrm {Pb}$ simulation are boosted by $\Delta y=\pm 0.465$ to account for the asymmetry of $\mathrm {p}$ and $\mathrm {Pb}$ beams in the laboratory frame. Samples for prompt and nonprompt $\mathrm{J}/{\psi }$ mesons are independently produced using the D6T [30] and Z2 [31] tunes, respectively. In the absence of experimental information on quarkonium polarization in $\mathrm {p}\mathrm {p}$ and $\mathrm {p}\mathrm {Pb}$ collisions at $\sqrt{s} =5.02\,\text {TeV}$, it is assumed that prompt $\mathrm{J}/{\psi }$ mesons are produced unpolarized, as observed in $\mathrm {p}\mathrm {p}$ collisions at $\sqrt{s} =7\,\text {TeV}$ [32–34]. The nonprompt $\mathrm{J}/{\psi }$ sample includes the polarization ($\lambda _{\theta }\approx -0.4$) determined from a measurement of the exclusive ${\mathrm {B}}$ hadron decays (${\mathrm {B}^{+}}, {\mathrm {B}^0}$, and $\mathrm{B}^0_\mathrm{s}$) as implemented in evtgen 9.1 [35]. The $\mathrm {p}\mathrm {Pb}$ measurements might be affected by physics processes with strong kinematic dependence within an analysis bin, e.g., polarization or energy loss. Such possible physics effects on the final cross sections are not included in the systematic uncertainties, as was done in the previous analyses [8, 9]. The QED final-state radiation from muons is simulated with photos 215.5 [36]. Finally, the CMS detector response is simulated using Geant4  [37].

## Analysis procedure

### Differential cross section, $R_{\mathrm {p}\mathrm {Pb}}$, and $R_{\mathrm {FB}}$

In this paper, three observables analyzed in $\mathrm{J}/{\psi }$ meson decays to muon pairs are reported. First, the cross sections are determined based on

2 $$\begin{aligned} \mathcal {B}(\mathrm{J}/{\psi } \rightarrow \mu ^+ \mu ^- )\frac{\mathrm{d}^2\sigma }{\mathrm{d}p_{\mathrm {T}} \,\mathrm{d}y_{\mathrm {CM}}} = \frac{N^{\mathrm{J}/{\psi }}_{\text {Fit}}/(\text {Acc}\,\varepsilon )}{\mathcal {L}_{\text {int}}\,\Delta p_{\mathrm {T}} \,\Delta y_{\mathrm {CM}}}, \end{aligned}$$

where $\mathcal {B}(\mathrm{J}/{\psi } \rightarrow \mu ^+ \mu ^- )$ is the branching fraction to the $\mu ^+ \mu ^-$ channel [38], $N^{\mathrm{J}/{\psi }}_{\text {Fit}}$ is the extracted raw yield of $\mathrm{J}/{\psi }$ mesons in a given $(p_{\mathrm {T}},y_{\mathrm {CM}})$ bin, $(\text {Acc}\,\varepsilon )$ represents the dimuon acceptance times efficiency described in Sect. 3.3, and $\mathcal {L}_{\text {int}}$ is the integrated luminosity with the values of $(28.0\pm 0.6)$ $\,\text {pb}^{-1}$ for $\mathrm {p}\mathrm {p}$  [39] and $(34.6\pm 1.2)$ $\,\text {nb}^{-1}$ for $\mathrm {p}\mathrm {Pb}$  [40] collisions.

The cross sections are measured in up to nine bins in $p_{\mathrm {T}}$ ([2,3], [3,4] [4,5], [5,6.5], [6.5,7.5], [7.5,8.5], [8.5,10], [10,14], [14,30]${\,\text {GeV}/{c}}$), with the minimum $p_{\mathrm {T}}$ values varying with $y_{\mathrm {CM}}$ ranges as shown in Table 1.

**Table 1:** Rapidity intervals and associated minimum $p_{\mathrm {T}}$ values for the $\mathrm{J}/{\psi }$ cross section measurements in $\mathrm {p}\mathrm {p}$ and $\mathrm {p}\mathrm {Pb}$ collisions

$y_{\mathrm {CM}}$	Minimum $p_{\mathrm {T}}$ (${\text {GeV}/{c}}$)	
	$\mathrm {p}\mathrm {p}$	$\mathrm {p}\mathrm {Pb}$
$1.93<y_{\mathrm {CM}} <2.4$	2	N/A
$1.5<y_{\mathrm {CM}} <1.93$	4	2
$0.9<y_{\mathrm {CM}} <1.5$	6.5	4
$0<y_{\mathrm {CM}} <0.9$	6.5	6.5
$-0.9<y_{\mathrm {CM}} <0$	6.5	6.5
$-1.5<y_{\mathrm {CM}} <-0.9$	6.5	6.5
$-1.93<y_{\mathrm {CM}} <-1.5$	4	5
$-2.4<y_{\mathrm {CM}} <-1.93$	2	4
$-2.87<y_{\mathrm {CM}} <-2.4$	N/A	2

The second observable considered is the nuclear modification factor, calculated as

3 $$\begin{aligned} R_{\mathrm {p}\mathrm {Pb}} (p_{\mathrm {T}},y_{\mathrm {CM}}) = \frac{({\mathrm{d}^2\sigma }/{\mathrm{d}p_{\mathrm {T}} \,\mathrm{d}y_{\mathrm {CM}}})_{\mathrm {p}\mathrm {Pb}}}{\mathrm {A}({\mathrm{d}^2\sigma }/{\mathrm{d}p_{\mathrm {T}} \,\mathrm{d}y_{\mathrm {CM}}})_{{\mathrm {p}\mathrm {p}}}}, \end{aligned}$$

where $\mathrm {A}=208$ is the number of nucleons in the $\mathrm {Pb}$ nucleus.

The third measurement is the forward-to-backward production ratio for $\mathrm {p}\mathrm {Pb}$ collisions, defined for positive $y_{\mathrm {CM}}$ by

4 $$\begin{aligned} R_{\mathrm {FB}} (p_{\mathrm {T}},y_{\mathrm {CM}} >0) = \frac{\mathrm{d}^2\sigma (p_{\mathrm {T}},y_{\mathrm {CM}})/\mathrm{d}p_{\mathrm {T}} \mathrm{d}y_{\mathrm {CM}}}{\mathrm{d}^2\sigma (p_{\mathrm {T}},-y_{\mathrm {CM}})/\mathrm{d}p_{\mathrm {T}} \mathrm{d}y_{\mathrm {CM}}}. \end{aligned}$$

This variable is a sensitive probe of the dynamics of $\mathrm{J}/{\psi }$ production by comparing nuclear effects in the forward and the backward $y_{\mathrm {CM}}$ hemispheres, since $R_{\mathrm {FB}} (p_{\mathrm {T}},y_{\mathrm {CM}})$ is equivalent to $R_{\mathrm {p}\mathrm {Pb}} (p_{\mathrm {T}},y_{\mathrm {CM}})/R_{\mathrm {p}\mathrm {Pb}} (p_{\mathrm {T}},-y_{\mathrm {CM}})$. In addition, several uncertainties cancel in the $R_{\mathrm {FB}}$ ratio, such as those from the integrated luminosity determination. The minimum $p_{\mathrm {T}}$ values for the $R_{\mathrm {FB}}$ measurement are 5${\,\text {GeV}/{c}}$ for $1.5<|y_{\mathrm {CM}} |<1.93$, and 6.5${\,\text {GeV}/{c}}$ for $|y_{\mathrm {CM}} |<1.5$. The ratio $R_{\mathrm {FB}}$ is also analyzed as a function of $E_{\mathrm {T}}^{{\mathrm {HF}}|\eta |>4}$, the transverse energy deposited on both sides of the collisions in the HF calorimeters within the $4<|\eta |<5.2$ range. This energy is related to the impact parameter of the collision. In Table 2, the mean value of $E_{\mathrm {T}}^{{\mathrm {HF}}|\eta |>4}$ and the fraction of events for each bin used in the analysis are computed from minimum bias $\mathrm {p}\mathrm {Pb}$ events.

**Table 2:** Ranges of forward transverse energy, $E_{\mathrm {T}}^{{\mathrm {HF}}|\eta |>4}$, their mean values, and associated fractions of $\mathrm {p}\mathrm {Pb}$ events that fall into each category

$E_{\mathrm {T}}^{{\mathrm {HF}}|\eta |>4} \,(\text {GeV})$	$\langle E_{\mathrm {T}}^{{\mathrm {HF}}|\eta |>4} \rangle$	Fraction (%)
0–20	9.4	73
20–30	24.3	18
>30	37.2	9

### Signal extraction

The signal extraction procedure is similar to that in previous CMS analyses of $\mathrm {p}\mathrm {p}$  [41, 42] and $\mathrm {PbPb}$  [6] collisions. The prompt $\mathrm{J}/{\psi }$ mesons are separated from those coming from ${\mathrm {B}}$ hadron decays by virtue of the pseudo-proper decay length, $\ell _{\mathrm{J}/{\psi }} =L_{xy}\,m_{\mathrm{J}/{\psi }}/p_{\mathrm {T}}$, where $L_{xy}$ is the transverse distance between the primary and secondary dimuon vertices in the laboratory frame, $m_{\mathrm{J}/{\psi }}$ is the mass of the $\mathrm{J}/{\psi }$ meson, and $p_{\mathrm {T}}$ is the dimuon transverse momentum. For each $p_{\mathrm {T}}$, $y_{\mathrm {CM}}$, and event activity bin, the fraction of nonprompt $\mathrm{J}/{\psi }$ mesons (*b fraction*) is evaluated through an extended unbinned maximum likelihood fit to the invariant mass spectrum and $\ell _{\mathrm{J}/{\psi }}$ distributions of $\mu ^+ \mu ^-$ pairs, sequentially. The invariant mass spectrum is fitted first, and some parameters are initialized and/or fixed. Then, the $\ell _{\mathrm{J}/{\psi }}$ distribution is fitted.

**Fig. 1:**
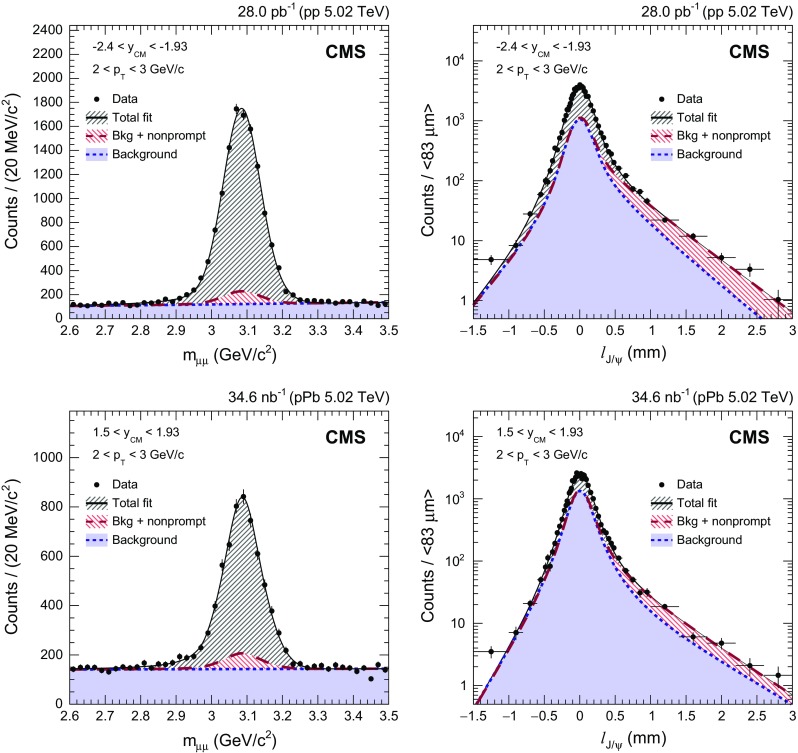
Examples of the invariant mass (*left*) and pseudo-proper decay length (*right*) distributions of $\mu ^+ \mu ^-$ pairs for $\mathrm {p}\mathrm {p}$ (*upper*) and $\mathrm {p}\mathrm {Pb}$ (*lower*) collisions. The bin widths of $\ell _{\mathrm{J}/{\psi }}$ distributions vary from 15 to 500 $\upmu$m, with the averaged value of 83 $\upmu$m. The projections of the 2D fit function onto the respective axes are overlaid as *solid lines*. The *long-dashed lines* show the fitted contribution of nonprompt $\mathrm{J}/{\psi }$ mesons. The fitted background contributions are shown by *short-dashed lines*

For the dimuon invariant mass distributions, the shape of the $\mathrm{J}/{\psi }$ signal is modeled by the sum of a Gaussian function and a Crystal Ball (CB) function [43], with common mean values and independent widths, in order to accommodate the rapidity-dependent mass resolution. The CB function combines a Gaussian core with a power-law tail using two parameters $n_{\mathrm {CB}}$ and $\alpha _{\mathrm {CB}}$, to describe final-state QED radiation of muons. Because the two parameters are strongly correlated, the value of $n_{\mathrm {CB}}$ is fixed at 2.1, while the $\alpha _{\mathrm {CB}}$ is a free parameter of the fit. This configuration gives the highest fit probability for data, in every $(p_{\mathrm {T}},y_{\mathrm {CM}})$ bin, when various settings of $\alpha _{\mathrm {CB}}$ and $n_{\mathrm {CB}}$ are tested. The invariant mass distribution of the underlying continuum background is represented by an exponential function.

For the $\ell _{\mathrm{J}/{\psi }}$ distributions, the prompt signal component is represented by a resolution function, which depends on the per-event uncertainty in the $\ell _{\mathrm{J}/{\psi }}$ provided by the reconstruction algorithm of primary and secondary vertices. The resolution function is composed of the sum of two Gaussian functions. A Gaussian with a narrower width ($\sigma _{\text {narrow}}$) describes the core of the signal component, while another with a greater width ($\sigma _{\text {wide}}$) accounts for the effect of uncertainties in the primary vertex determination and has a fixed value based on MC simulations. The $\ell _{\mathrm{J}/{\psi }}$ distribution of the nonprompt component is modeled by an exponential decay function convolved with a resolution function. The continuum background component is modeled by the sum of three exponential decay functions, a normal one on one side $\ell _{\mathrm{J}/{\psi }} >0$, a flipped one on the other side $\ell _{\mathrm{J}/{\psi }} <0$, and a double-sided one, which are also convolved with a resolution function. The parameters describing the $\ell _{\mathrm{J}/{\psi }}$ distributions of the background are determined from sidebands in the invariant mass distribution $2.6<m_{\mu \mu }<2.9{\,\text {GeV}/c^{2}}$ and $3.3<m_{\mu \mu }<3.5{\,\text {GeV}/c^{2}}$. The results are insensitive to the selection of sideband ranges.

For $\mathrm {p}\mathrm {Pb}$ analysis, two data sets corresponding to each beam direction are merged and fitted together, after it is determined that the results are compatible with those from a separate analysis, performed over each data set. Figure 1 shows examples of fit projections onto the mass (left) and $\ell _{\mathrm{J}/{\psi }}$ (right) axes for muon pairs with $2<p_{\mathrm {T}} <3{\,\text {GeV}/{c}}$ in $-2.4<y_{\mathrm {CM}} <-1.93$ from $\mathrm {p}\mathrm {p}$ (upper), and in $1.5<y_{\mathrm {CM}} <1.93$ from $\mathrm {p}\mathrm {Pb}$ (lower) collisions.

### Corrections

The acceptance and reconstruction, identification, and trigger efficiency corrections are evaluated from the MC simulation described in Sect. 2. The acceptance is estimated by the fraction of generated $\mathrm{J}/{\psi }$ mesons in each $(p_{\mathrm {T}},y_{\mathrm {CM}})$ bin, decaying into two muons, each within the fiducial phase space defined in Eq. (1).

In order to compensate for imperfections in the simulation-based efficiencies, an additional scaling factor is applied, calculated with a *tag-and-probe* (T&P) method [44]. The tag muons require tight identification, and the probe muons are selected with and without satisfying the selection criteria relevant to the efficiency being measured. Then, invariant mass distributions of tag and probe pairs in the $\mathrm{J}/{\psi }$ mass range are fitted to count the number of signals in the two groups. The single-muon efficiencies are deduced from the ratio of $\mathrm{J}/{\psi }$ mesons in the passing-probe over all-probe group. The data-to-simulation ratios of single-muon efficiencies are used to correct the dimuon efficiencies, taking the kinematic distributions of decayed muons into account. The dimuon efficiency weights evaluated by the T&P method are similar for $\mathrm {p}\mathrm {p}$ and $\mathrm {p}\mathrm {Pb}$ events and range from 0.98 to 1.90, with the largest one coming from the lowest $p_{\mathrm {T}}$ bin. The efficiencies are independent of the event activity, as verified by $\mathrm {p}\mathrm {Pb}$ data and in a pythia sample embedded in simulated $\mathrm {p}\mathrm {Pb}$ events generated by hijing 1.383 [45].

In addition, the shape of the uncorrected distributions of $\mathrm{J}/{\psi }$ yield versus $p_{\mathrm {T}}$ in data and MC samples are observed to be different. To resolve the possible bias in acceptance and efficiency corrections, the data-to-simulation ratios are fitted by empirical functions and used to reweight the $p_{\mathrm {T}}$ spectra in MC samples for each $y_{\mathrm {CM}}$ bin. The effect of reweighting on the acceptance and efficiency is detailed in the next Section.

### Systematic uncertainties

The following sources of systematic uncertainties are considered: fitting procedure, acceptance and efficiency corrections, and integrated luminosities.

To estimate the systematic uncertainty due to the fitting procedure, variations of the parameters or alternative fit functions have been considered for the invariant mass and $\ell _{\mathrm{J}/{\psi }}$ distributions. For the signal shape in the invariant mass distributions, three alternative parameter settings are tested: (1) $\alpha _{\mathrm {CB}}$ is set to 1.7, averaged from the default fit, and $n_{\mathrm {CB}}$ free, (2) both $\alpha _{\mathrm {CB}}$ and $n_{\mathrm {CB}}$ are left free, and (3) both are obtained from a MC template and then fixed when fit to the data. The maximum deviation of yields among these three variations is quoted as the uncertainty. For the background fit of the invariant mass distributions, a first-order polynomial is used as an alternative. For the shape of $\ell _{\mathrm{J}/{\psi }}$ distribution of prompt $\mathrm{J}/{\psi }$ mesons, two alternatives are studied: (1) both $\sigma _{\text {wide}}$ and $\sigma _{\text {narrow}}$ are left free, and (2) both parameters are fixed to the MC templates. The maximum deviation of yields is taken as the uncertainty. Finally, for the $\ell _{\mathrm{J}/{\psi }}$ distribution shape of nonprompt $\mathrm{J}/{\psi }$ mesons, the template shape is directly taken from reconstructed MC events. The uncertainties from the previously mentioned methods are 0.7–5.0% for prompt and 1.1–36.3% for nonprompt $\mathrm{J}/{\psi }$ mesons. They are larger for the shape variations in the $\ell _{\mathrm{J}/{\psi }}$ than in the invariant mass distributions, especially for nonprompt $\mathrm{J}/{\psi }$ mesons.

For the uncertainties from acceptance and efficiency correction factors, the effect of reweighting the $p_{\mathrm {T}}$ spectrum of events generated by pythia generator as described in Sect. 3.3 is considered. The deviation of the correction factors obtained from the default pythia spectra and those from data-based weighted spectra is less than 2.9% across all kinematic ranges. The full deviation values are quoted as the systematic uncertainties. The determination of uncertainties for T&P corrections is performed by propagating the uncertainties in single-muon efficiencies to the dimuon efficiency values. The systematic uncertainties are evaluated by varying the fit conditions in the T&P procedure, and the statistical uncertainties are estimated using a fast parametric simulation. The total uncertainty from T&P corrections is obtained by the quadratic sum of two sources. Uncertainties from the efficiency correction, including the T&P uncertainties, range from 2.4 to 6.1%, and tend to be larger for lower $p_{\mathrm {T}}$. The uncertainty in the integrated luminosities (2.3% for $\mathrm {p}\mathrm {p}$  [39] and 3.5% for $\mathrm {p}\mathrm {Pb}$  [40]) is correlated across all data points and affects only the production cross sections and $R_{\mathrm {p}\mathrm {Pb}}$, while it cancels out in the $R_{\mathrm {FB}}$ measurements.

Table 3 summarizes systematic uncertainties considered in this analysis. The range refers to different $(p_{\mathrm {T}},y_{\mathrm {CM}})$ bins; the uncertainties tend to be lower at high $p_{\mathrm {T}}$ and midrapidity, and higher at low $p_{\mathrm {T}}$ and forward or backward $y_{\mathrm {CM}}$. The larger uncertainties of the nonprompt $\mathrm{J}/{\psi }$ yields come from the signal extraction in their lowest $p_{\mathrm {T}}$ bin, 2–3${\,\text {GeV}/{c}}$. In the case of the $R_{\mathrm {p}\mathrm {Pb}}$ measurements with a $p_{\mathrm {T}}$ limit of 4${\,\text {GeV}/{c}}$, maximum uncertainties for nonprompt $\mathrm{J}/{\psi }$ mesons are 12.7% for $\mathrm {p}\mathrm {p}$ and 12.8% for $\mathrm {p}\mathrm {Pb}$ collisions. The total systematic uncertainty is evaluated as the quadratic sum of the uncertainties from all sources in each kinematic bin, except for those from the integrated luminosity determination.

**Table 3:** Summary of the relative systematic uncertainties for the cross section measurements, given in percentages, for prompt and nonprompt $\mathrm{J}/{\psi }$ mesons in $\mathrm {p}\mathrm {p}$ and $\mathrm {p}\mathrm {Pb}$ collisions

	Prompt $\mathrm{J}/{\psi }$		Nonprompt $\mathrm{J}/{\psi }$	
	$\mathrm {p}\mathrm {p}$	$\mathrm {p}\mathrm {Pb}$	$\mathrm {p}\mathrm {p}$	$\mathrm {p}\mathrm {Pb}$
Signal extraction	0.8–3.2	0.7–5.0	2.0–36.3	1.1–29.5
Efficiency	2.4–4.4	2.4–6.1	2.4–4.3	2.4–6.1
Acceptance	0.0–2.3	0.0–1.2	0.0–1.3	0.0–1.3
Integrated luminosity	2.3	3.5	2.3	3.5
Total	2.7–5.3	2.8–7.1	3.4–36.5	3.3–30.1

## Results

### Prompt $\mathrm{J}/{\psi }$ mesons

**Fig. 2:**
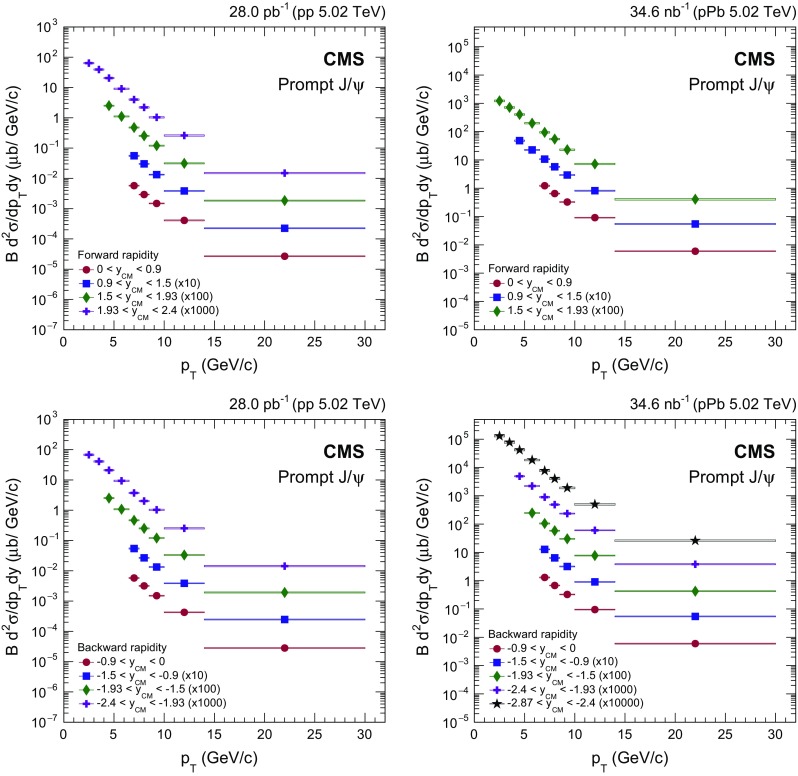
Differential cross section (multiplied by the dimuon branching fraction) of prompt $\mathrm{J}/{\psi }$ mesons in $\mathrm {p}\mathrm {p}$ (*left*) and $\mathrm {p}\mathrm {Pb}$ (*right*) collisions at forward (*upper*) and backward (*lower*) $y_{\mathrm {CM}}$. The *vertical bars* (smaller than the *symbols* in most cases) represent the statistical uncertainties and the *shaded boxes* show the systematic uncertainties. The fully correlated global uncertainty from the integrated luminosity determination, 2.3% for $\mathrm {p}\mathrm {p}$ and 3.5% for $\mathrm {p}\mathrm {Pb}$ collisions, is not included in the point-by-point uncertainties

**Fig. 3:**
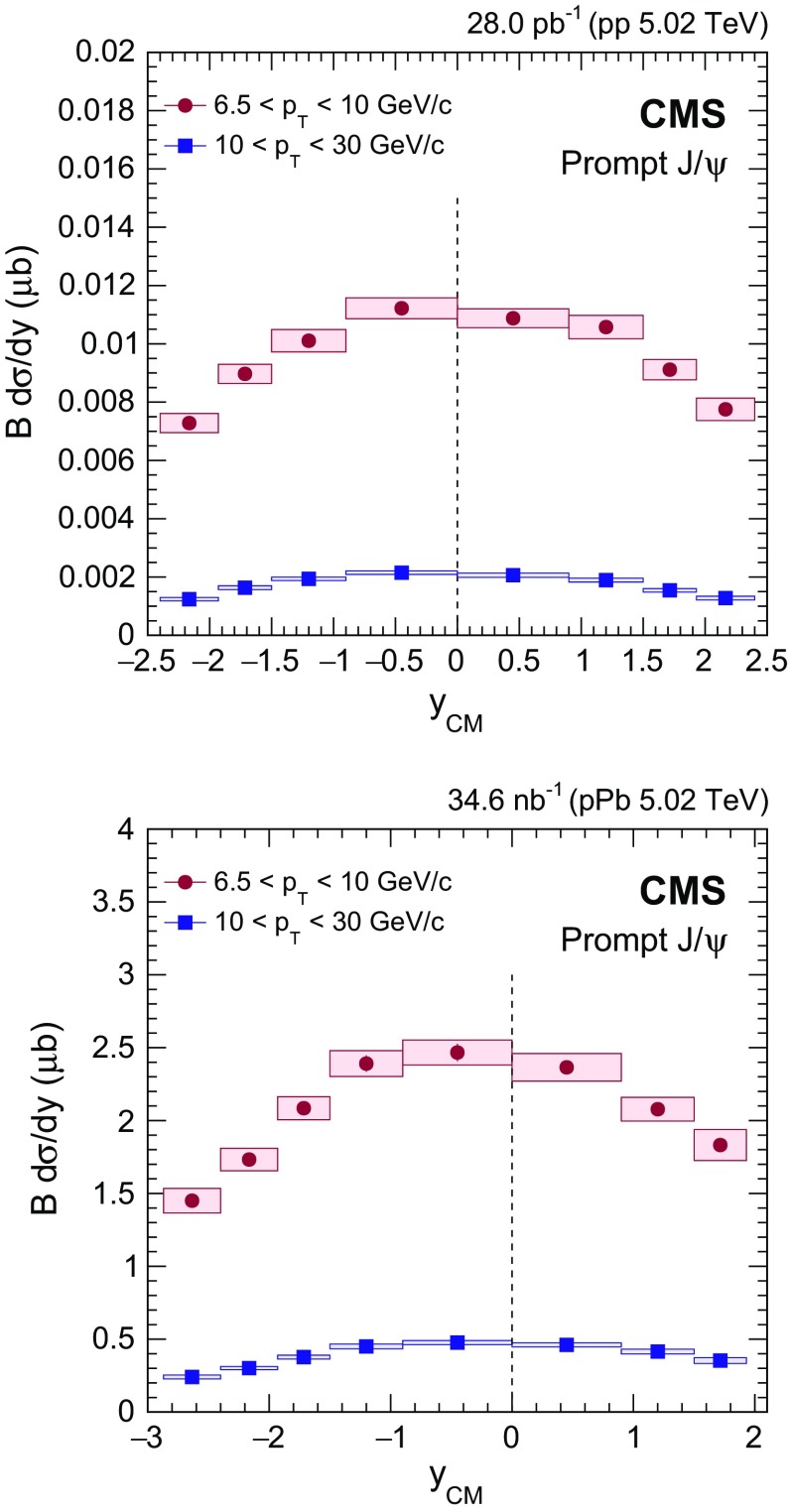
Rapidity dependence of the cross section (multiplied by the dimuon branching fraction) for prompt $\mathrm{J}/{\psi }$ mesons in the $p_{\mathrm {T}}$ intervals of $6.5<p_{\mathrm {T}} <10{\,\text {GeV}/{c}}$ (*circles*) and $10<p_{\mathrm {T}} <30{\,\text {GeV}/{c}}$ (*squares*) in $\mathrm {p}\mathrm {p}$ (*upper*) and $\mathrm {p}\mathrm {Pb}$ (*lower*) collisions. The *vertical dashed line* indicates $y_{\mathrm {CM}} =0$. The *vertical bars* (smaller than the *symbols* in most cases) represent the statistical uncertainties and the *shaded boxes* show the systematic uncertainties. The fully correlated global uncertainty from the integrated luminosity determination, 2.3% for $\mathrm {p}\mathrm {p}$ and 3.5% for $\mathrm {p}\mathrm {Pb}$ collisions, is not included in the point-by-point uncertainties

Figure 2 shows the double-differential prompt $\mathrm{J}/{\psi }$ production cross sections multiplied by the dimuon branching fraction in $\mathrm {p}\mathrm {p}$ (left) and $\mathrm {p}\mathrm {Pb}$ (right) collisions, with data points plotted at the center of each bin. Statistical uncertainties are displayed as vertical bars, while boxes that span the $p_{\mathrm {T}}$ bin width represent systematic uncertainties. Not shown is a global normalization uncertainty of 2.3% in $\mathrm {p}\mathrm {p}$ and 3.5% in $\mathrm {p}\mathrm {Pb}$ collisions arising from the integrated luminosity determination.

Prompt $\mathrm{J}/{\psi }$ $y_{\mathrm {CM}}$ distributions are shown in Fig. 3 in $\mathrm {p}\mathrm {p}$ (upper) and $\mathrm {p}\mathrm {Pb}$ (lower) collisions. The measurements are integrated over two $p_{\mathrm {T}}$ intervals, $6.5<p_{\mathrm {T}} <10{\,\text {GeV}/{c}}$ (low $p_{\mathrm {T}}$) and $10<p_{\mathrm {T}} <30{\,\text {GeV}/{c}}$ (high $p_{\mathrm {T}}$).

**Fig. 4:**
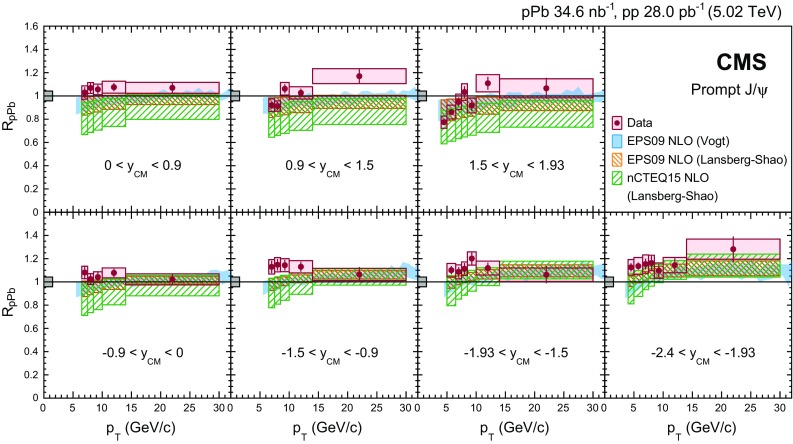
Transverse momentum dependence of $R_{\mathrm {p}\mathrm {Pb}}$ for prompt $\mathrm{J}/{\psi }$ mesons in seven $y_{\mathrm {CM}}$ ranges. The *vertical bars* represent the statistical uncertainties and the *shaded boxes* show the systematic uncertainties. The fully correlated global uncertainty of 4.2% is displayed as a *gray box* at $R_{\mathrm {p}\mathrm {Pb}} =1$ next to the *left axis*. The predictions of shadowing models based on the parameterizations EPS09 and nCTEQ15 [14, 46–48] are also shown

The $p_{\mathrm {T}}$ dependence of prompt $\mathrm{J}/{\psi }$ $R_{\mathrm {p}\mathrm {Pb}}$ is shown in Fig. 4, in seven $y_{\mathrm {CM}}$ ranges for which $\mathrm {p}\mathrm {p}$ and $\mathrm {p}\mathrm {Pb}$ measurements overlap. Around midrapidity ($|y_{\mathrm {CM}} |<0.9$) and in the three backward $y_{\mathrm {CM}}$ bins (lower panels), $R_{\mathrm {p}\mathrm {Pb}}$ is slightly above unity without a clear dependence on $p_{\mathrm {T}}$. In the most forward bin ($1.5<y_{\mathrm {CM}} <1.93$), suppression at low $p_{\mathrm {T}}$ (${\lesssim }7.5{\,\text {GeV}/{c}}$) is observed, followed by a weak increase of $R_{\mathrm {p}\mathrm {Pb}}$ at higher $p_{\mathrm {T}}$. The results are compared to three model calculations. One is based on the next-to-leading order (NLO) Color Evaporation Model [14] using the EPS09 [46] nPDF set. The other two are calculated from the nPDF sets of EPS09 and nCTEQ15 [47], respectively, with the parameterization of $2\rightarrow 2$ partonic scattering process based on data, as described in Ref. [48]. All three $R_{\mathrm {p}\mathrm {Pb}}$ calculations are marginally lower than the measured values across all $y_{\mathrm {CM}}$ bins. The calculations based on coherent energy loss are not yet available to describe quarkonium production at large $p_{\mathrm {T}}$ (${\gtrsim } m_{\mathrm{J}/{\psi }}$); therefore, no comparison of the present data with the model [15] is performed.

It is worth noting that the $R_{\mathrm {p}\mathrm {Pb}}$ values measured in the most forward ($1.5<y_{\mathrm {CM}} <1.93$) and backward ($-2.4<y_{\mathrm {CM}} <-1.93$) regions are consistent, in the overlapping $p_{\mathrm {T}}$ intervals ($4<p_{\mathrm {T}} <8{\,\text {GeV}/{c}}$), with the inclusive $\mathrm{J}/{\psi }$ results of the ALICE collaboration [21, 22] over $2.03<y_{\mathrm {CM}} <3.53$ and $-4.46<y_{\mathrm {CM}} <-2.96$, obtained using an interpolated $\mathrm {p}\mathrm {p}$ cross section reference. Although the ALICE results are for inclusive $\mathrm{J}/{\psi }$ mesons, the nonprompt contribution is expected to be relatively small (${<}20\%$) in the domain $p_{\mathrm {T}} <8{\,\text {GeV}/{c}}$.

**Fig. 5:**
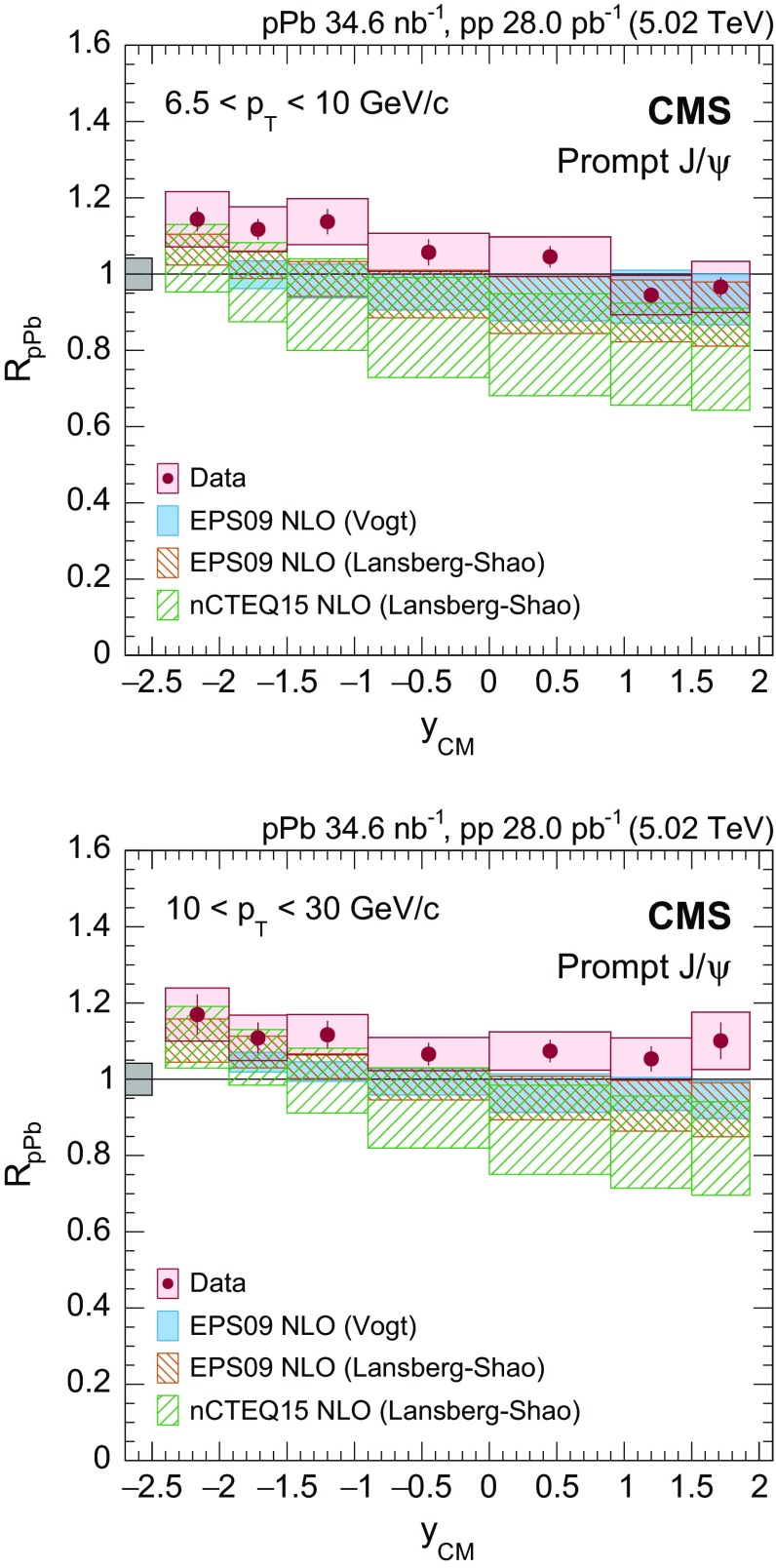
Rapidity dependence of $R_{\mathrm {p}\mathrm {Pb}}$ for prompt $\mathrm{J}/{\psi }$ mesons in two $p_{\mathrm {T}}$ ranges: $6.5<p_{\mathrm {T}} <10{\,\text {GeV}/{c}}$ (*upper*) and $10<p_{\mathrm {T}} <30{\,\text {GeV}/{c}}$ (*lower*). The *vertical bars* represent the statistical uncertainties and the *shaded boxes* show the systematic uncertainties. The fully correlated global uncertainty of 4.2% is displayed as a *gray box* at $R_{\mathrm {p}\mathrm {Pb}} =1$ next to the *left axis*. The predictions of shadowing models based on the parameterizations EPS09 and nCTEQ15 [14, 46–48] are also shown

Figure 5 displays the $y_{\mathrm {CM}}$ dependence of prompt $\mathrm{J}/{\psi }$ $R_{\mathrm {p}\mathrm {Pb}}$ in the low-$p_{\mathrm {T}}$ (upper) and the high-$p_{\mathrm {T}}$ (lower) regions corresponding to the same $p_{\mathrm {T}}$ bins used in Fig. 3. In the high-$p_{\mathrm {T}}$ region, $R_{\mathrm {p}\mathrm {Pb}}$ is above unity over the whole $y_{\mathrm {CM}}$ range. In the lower-$p_{\mathrm {T}}$ region, a decrease of $R_{\mathrm {p}\mathrm {Pb}}$ for increasing $y_{\mathrm {CM}}$ is suggested. The same theoretical predictions shown in Fig. 4 are overlaid. In contrast to the measurement of $\mathrm{J}/{\psi }$ mesons in $\mathrm {PbPb}$ collisions [6], no significant deviation from unity is observed in the $p_{\mathrm {T}}$ and $y_{\mathrm {CM}}$ ranges studied here. This suggests that the strong suppression of $\mathrm{J}/{\psi }$ production in $\mathrm {PbPb}$ collisions is an effect of QGP formation.

**Fig. 6:**
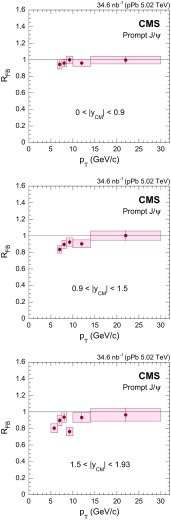
Transverse momentum dependence of $R_{\mathrm {FB}}$ for prompt $\mathrm{J}/{\psi }$ mesons in three $y_{\mathrm {CM}}$ regions. The *vertical bars* represent the statistical uncertainties and the *shaded boxes* show the systematic uncertainties

The forward-to-backward ratio of $\mathrm {p}\mathrm {Pb}$ cross sections, $R_{\mathrm {FB}}$, in three $y_{\mathrm {CM}}$ ranges is displayed as a function of $p_{\mathrm {T}}$ for prompt $\mathrm{J}/{\psi }$ mesons in Fig. 6. The $R_{\mathrm {FB}}$ tends to be below unity at low $p_{\mathrm {T}} \lesssim 7.5{\,\text {GeV}/{c}}$ and forward $|y_{\mathrm {CM}} |>0.9$. In the $6.5<p_{\mathrm {T}} <10{\,\text {GeV}/{c}}$ bin, an indication of decrease of $R_{\mathrm {FB}}$ with increasing $y_{\mathrm {CM}}$ is observed. The results are in agreement with the measurements from the ATLAS [23], ALICE [21, 22], and LHCb [24] collaborations.

**Fig. 7:**
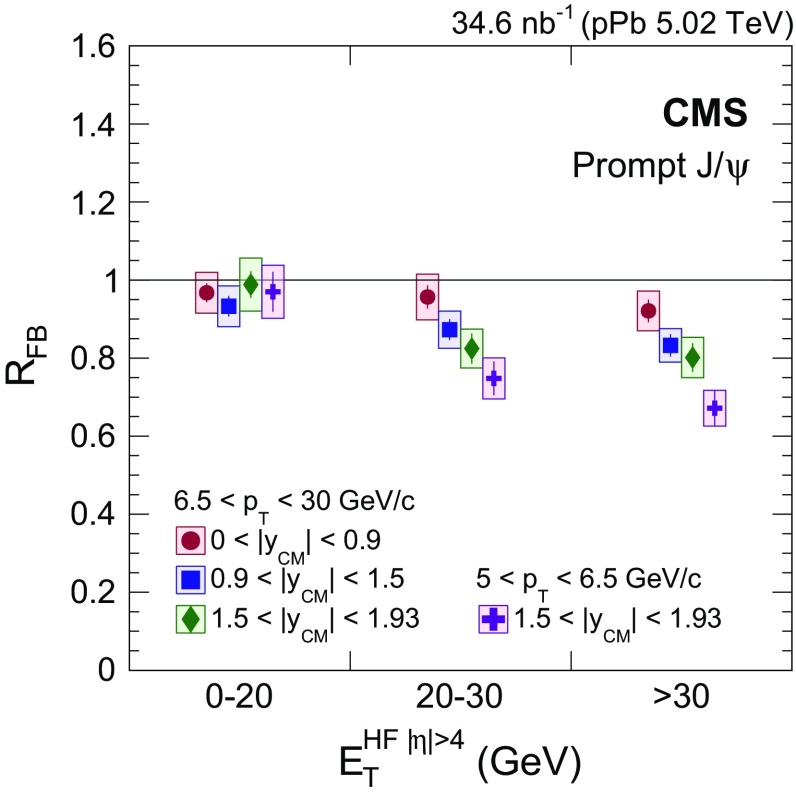
Dependence of $R_{\mathrm {FB}}$ for prompt $\mathrm{J}/{\psi }$ mesons on the hadronic activity in the event, given by the transverse energy deposited in the CMS detector at large pseudorapidities $E_{\mathrm {T}}^{{\mathrm {HF}}|\eta |>4}$. Data points are slightly shifted horizontally so that they do not overlap. The *vertical bars* represent the statistical uncertainties and the *shaded boxes* show the systematic uncertainties

Figure 7 shows $R_{\mathrm {FB}}$ as a function of $E_{\mathrm {T}}^{{\mathrm {HF}}|\eta |>4}$ for prompt $\mathrm{J}/{\psi }$ mesons in three $y_{\mathrm {CM}}$ ranges. The data are integrated over $6.5<p_{\mathrm {T}} <30{\,\text {GeV}/{c}}$; a lower-$p_{\mathrm {T}}$ bin, $5<p_{\mathrm {T}} <6.5{\,\text {GeV}/{c}}$, is shown in addition for the most forward-backward interval, $1.5<|y_{\mathrm {CM}} |<1.93$. The value of $R_{\mathrm {FB}}$ decreases as a function of $E_{\mathrm {T}}^{{\mathrm {HF}}|\eta |>4}$, suggesting that the effects that cause the asymmetry between the forward-to-backward production are larger in events with more hadronic activity.

### Nonprompt $\mathrm{J}/{\psi }$ mesons

**Fig. 8:**
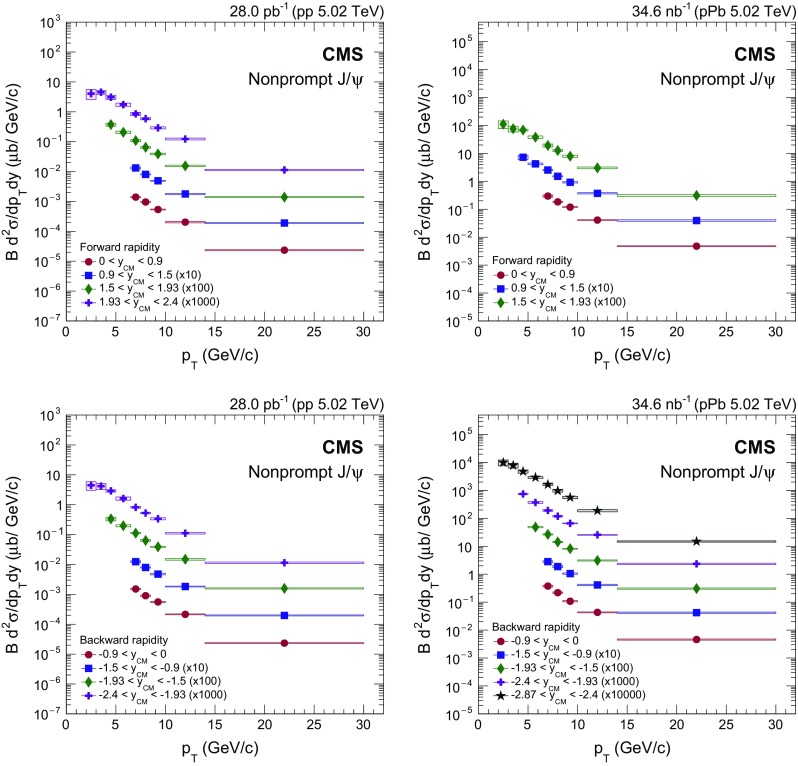
Differential cross section (multiplied by the dimuon branching fraction) of nonprompt $\mathrm{J}/{\psi }$ mesons in $\mathrm {p}\mathrm {p}$ (*left*) and $\mathrm {p}\mathrm {Pb}$ (*right*) collisions at forward (*upper*) and backward (*lower*) $y_{\mathrm {CM}}$. The *vertical bars* (smaller than the *symbols* in most cases) represent the statistical uncertainties and the *shaded boxes* show the systematic uncertainties. The fully correlated global uncertainty from the integrated luminosity determination, 2.3% for $\mathrm {p}\mathrm {p}$ and 3.5% for $\mathrm {p}\mathrm {Pb}$ collisions, is not included in the point-by-point uncertainties

**Fig. 9:**
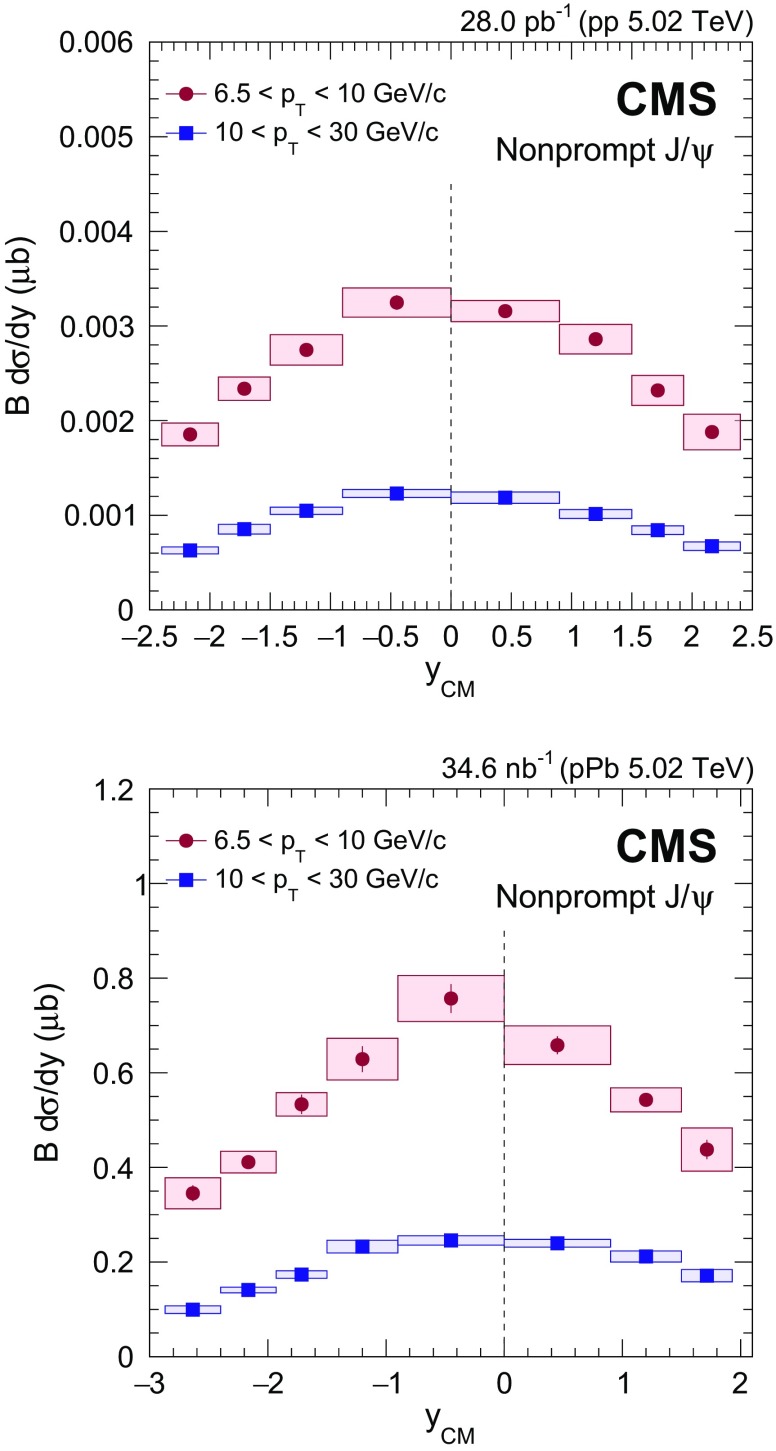
Rapidity dependence of the cross section (multiplied by the dimuon branching fraction) for nonprompt $\mathrm{J}/{\psi }$ mesons in the $p_{\mathrm {T}}$ intervals of $6.5<p_{\mathrm {T}} <10{\,\text {GeV}/{c}}$ (*circles*) and $10<p_{\mathrm {T}} <30{\,\text {GeV}/{c}}$ (*squares*) in $\mathrm {p}\mathrm {p}$ (*upper*) and $\mathrm {p}\mathrm {Pb}$ (*lower*) collisions. The *vertical dashed line* indicates $y_{\mathrm {CM}} =0$. The *vertical bars* (smaller than the *symbols* in most cases) represent the statistical uncertainties and the *shaded boxes* show the systematic uncertainties. The fully correlated global uncertainty from the integrated luminosity determination, 2.3% for $\mathrm {p}\mathrm {p}$ and 3.5% for $\mathrm {p}\mathrm {Pb}$ collisions, is not included in the point-by-point uncertainties

**Fig. 10:**
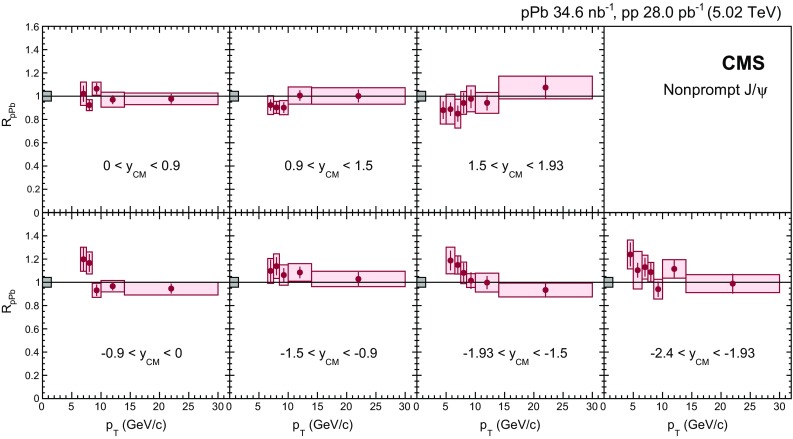
Transverse momentum dependence of $R_{\mathrm {p}\mathrm {Pb}}$ for nonprompt $\mathrm{J}/{\psi }$ mesons in seven $y_{\mathrm {CM}}$ ranges. The *vertical bars* represent the statistical uncertainties and the *shaded boxes* show the systematic uncertainties. The fully correlated global uncertainty of 4.2% is displayed as a *gray box* at $R_{\mathrm {p}\mathrm {Pb}} =1$ next to the *left axis*

**Fig. 11:**
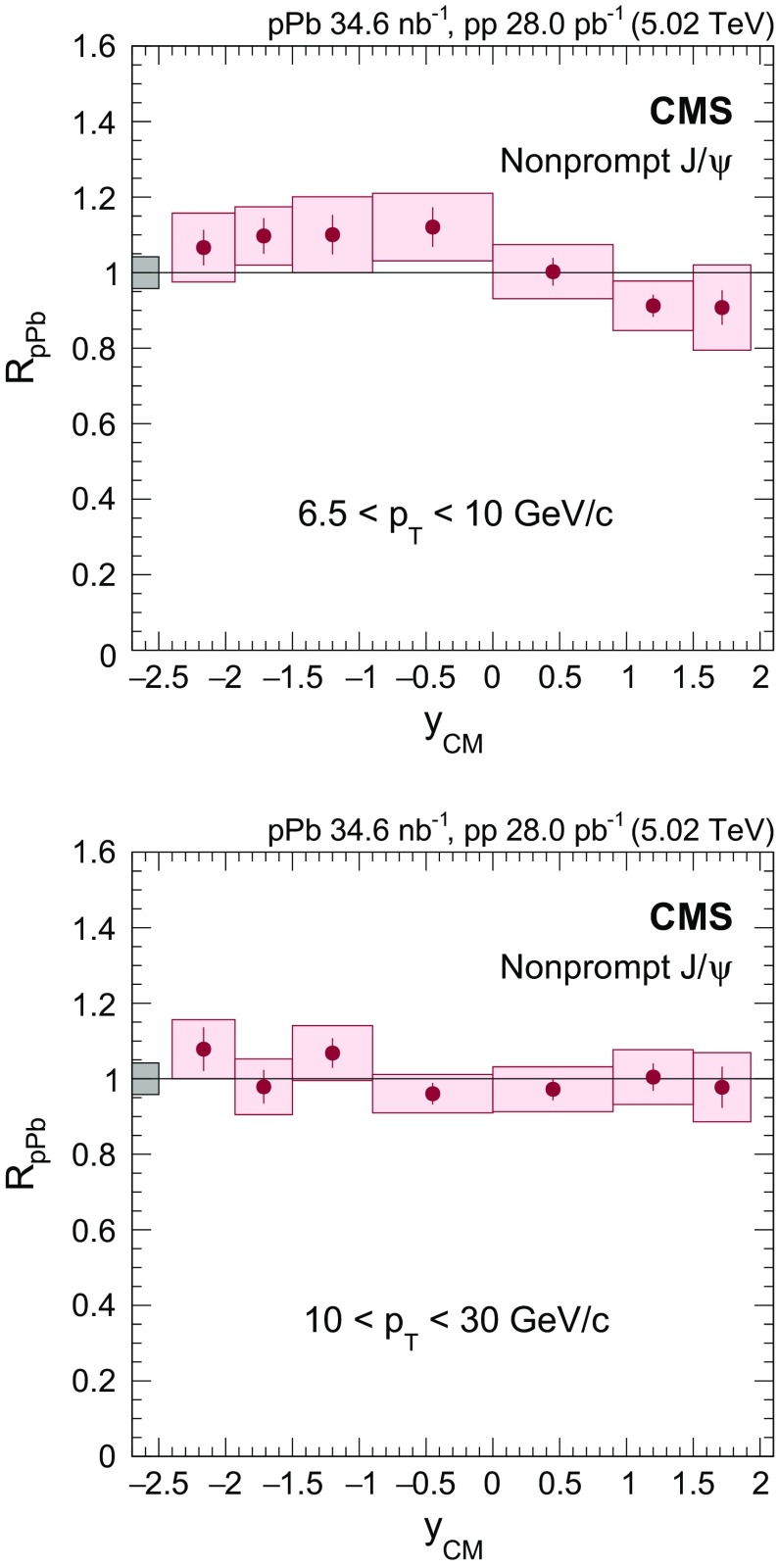
Rapidity dependence of $R_{\mathrm {p}\mathrm {Pb}}$ for nonprompt $\mathrm{J}/{\psi }$ mesons in two $p_{\mathrm {T}}$ ranges: $6.5<p_{\mathrm {T}} <10{\,\text {GeV}/{c}}$ (*upper*) and $10<p_{\mathrm {T}} <30{\,\text {GeV}/{c}}$ (*lower*). The *vertical bars* represent the statistical uncertainties and the *shaded boxes* show the systematic uncertainties. The fully correlated global uncertainty of 4.2% is displayed as a *gray box* at $R_{\mathrm {p}\mathrm {Pb}} =1$ next to the *left axis*

**Fig. 12:**
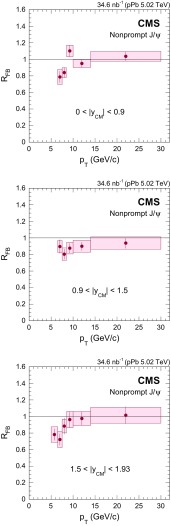
Transverse momentum dependence of $R_{\mathrm {FB}}$ for nonprompt $\mathrm{J}/{\psi }$ mesons in three $y_{\mathrm {CM}}$ regions. The *vertical bars* represent the statistical uncertainties and the *shaded boxes* show the systematic uncertainties

**Fig. 13:**
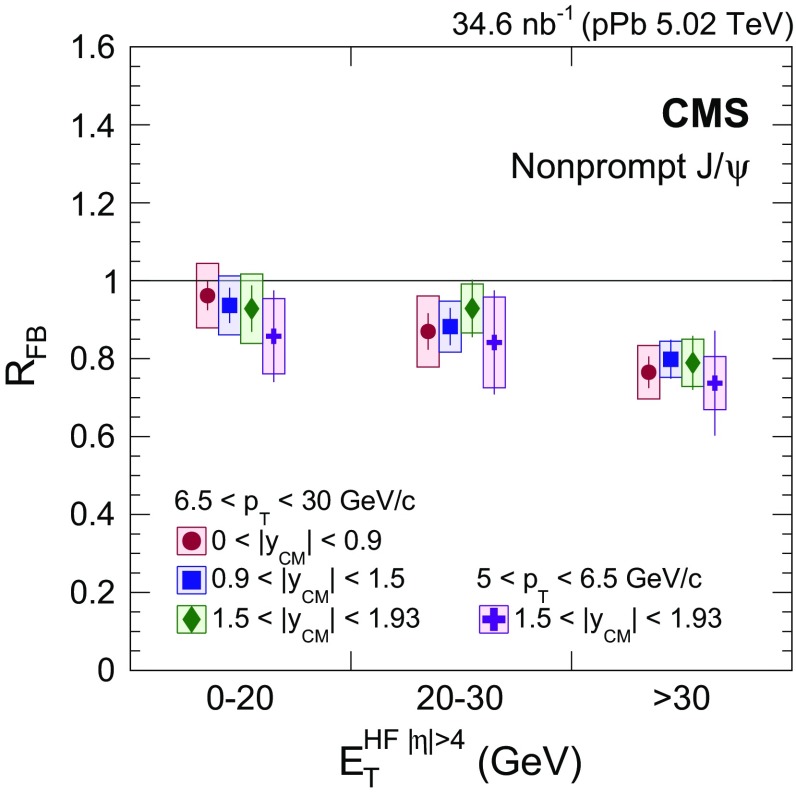
Dependence of $R_{\mathrm {FB}}$ for nonprompt $\mathrm{J}/{\psi }$ mesons on the hadronic activity in the event, given by the transverse energy deposited in the CMS detector at large pseudorapidities $E_{\mathrm {T}}^{{\mathrm {HF}}|\eta |>4}$. Data points are slightly shifted horizontally so that they do not overlap. The *vertical bars* represent the statistical uncertainties and the *shaded boxes* show the systematic uncertainties

The same distributions and observables discussed in Sect. 4.1 have been investigated for the nonprompt $\mathrm{J}/{\psi }$ meson samples. Differential cross sections are plotted as functions of $p_{\mathrm {T}}$ and $y_{\mathrm {CM}}$ in Figs. 8 and 9, respectively, using the same binning as for prompt $\mathrm{J}/{\psi }$ mesons.

The measurement of $R_{\mathrm {p}\mathrm {Pb}}$ for nonprompt $\mathrm{J}/{\psi }$ mesons shown in Fig. 10 as a function of $p_{\mathrm {T}}$ is compatible with unity in all $y_{\mathrm {CM}}$ bins. The somewhat larger uncertainties, however, make it difficult to draw firm conclusions for the nonprompt $\mathrm{J}/{\psi }$ production. The $y_{\mathrm {CM}}$ dependence of nonprompt $\mathrm{J}/{\psi }$ $R_{\mathrm {p}\mathrm {Pb}}$ integrated in the low- and high-$p_{\mathrm {T}}$ regions is shown in Fig. 11. In all $y_{\mathrm {CM}}$ bins, $R_{\mathrm {p}\mathrm {Pb}}$ is consistent with unity although the data hint at a rapidity dependence for $R_{\mathrm {p}\mathrm {Pb}}$ in the low $p_{\mathrm {T}}$ region, as found in the prompt $\mathrm{J}/{\psi }$ meson production (Fig. 5).

Figures 12 and 13 show the $p_{\mathrm {T}}$ and $E_{\mathrm {T}}^{{\mathrm {HF}}|\eta |>4}$ dependence of nonprompt $\mathrm{J}/{\psi }$ $R_{\mathrm {FB}}$, respectively. The $R_{\mathrm {FB}}$ ratios seem to increase slightly with $p_{\mathrm {T}}$ from ${\sim }0.8\pm 0.1$ to ${\sim }1.0\pm 0.1$ in all $y_{\mathrm {CM}}$ bins. The results are consistent with those from the ATLAS [23] and LHCb [24] collaborations within uncertainties. As seen for prompt $\mathrm{J}/{\psi }$ meson production, $R_{\mathrm {FB}}$ for nonprompt $\mathrm{J}/{\psi }$ meson production decreases with $E_{\mathrm {T}}^{{\mathrm {HF}}|\eta |>4}$, indicating the presence of different nuclear effects at forward than at backward $y_{\mathrm {CM}}$ in the regions with the greatest event activity.

## Summary

Proton–proton ($\mathrm {p}\mathrm {p}$) and proton–lead ($\mathrm {p}\mathrm {Pb}$) data at $\sqrt{s_{\mathrm {NN}}} =5.02\,\text {TeV}$ collected with the CMS detector are used to investigate the production of prompt and nonprompt $\mathrm{J}/{\psi }$ mesons and its possible modification due to cold nuclear matter effects. Double-differential cross sections, as well as the nuclear modification factor $R_{\mathrm {p}\mathrm {Pb}}$ and forward-to-backward production ratio $R_{\mathrm {FB}}$, are reported as functions of the $\mathrm{J}/{\psi }$ $p_{\mathrm {T}}$ and $y_{\mathrm {CM}}$.

The $R_{\mathrm {p}\mathrm {Pb}}$ values for prompt $\mathrm{J}/{\psi }$ mesons are above unity in mid- and backward $y_{\mathrm {CM}}$ intervals analyzed ($-2.4<y_{\mathrm {CM}} <0.9$), with a possible depletion in the most forward bin at low $p_{\mathrm {T}} \lesssim 7.5{\,\text {GeV}/{c}}$. In the case of nonprompt $\mathrm{J}/{\psi }$ meson production, $R_{\mathrm {p}\mathrm {Pb}}$ is compatible with unity in all $y_{\mathrm {CM}}$ bins. The prompt $\mathrm{J}/{\psi }$ $R_{\mathrm {FB}}$ is below unity for $p_{\mathrm {T}} \lesssim 7.5{\,\text {GeV}/{c}}$ and forward $|y_{\mathrm {CM}} |>0.9$, but is consistent with unity for $p_{\mathrm {T}} \gtrsim 10{\,\text {GeV}/{c}}$. For nonprompt $\mathrm{J}/{\psi }$ mesons, $R_{\mathrm {FB}}$ tends to be below unity at $p_{\mathrm {T}} \lesssim 7.5{\,\text {GeV}/{c}}$ and increases for higher $p_{\mathrm {T}}$, but with slightly larger uncertainties. The dependence of $R_{\mathrm {FB}}$ on the hadronic activity in $\mathrm {p}\mathrm {Pb}$ events has been studied through the variable $E_{\mathrm {T}}^{{\mathrm {HF}}|\eta |>4}$, characterizing the transverse energy deposited in the CMS detector at large pseudorapidities $4<|\eta |<5.2$. The $R_{\mathrm {FB}}$ ratio is observed to decrease with increasing event activity for both prompt and nonprompt $\mathrm{J}/{\psi }$ mesons, indicating enhanced nuclear matter effects for increasingly central $\mathrm {p}\mathrm {Pb}$ collisions.

A depletion of prompt $\mathrm{J}/{\psi }$ mesons in $\mathrm {p}\mathrm {Pb}$ collisions (as compared to $\mathrm {p}\mathrm {p}$ collisions) is expected in the forward $y_{\mathrm {CM}}$ region because of the shadowing of nuclear parton distributions and/or coherent energy loss effects. Such a suppression is observed in the measurements presented in this paper at $y_{\mathrm {CM}} >1.5$ and $p_{\mathrm {T}} \lesssim 7.5{\,\text {GeV}/{c}}$, but not at larger $p_{\mathrm {T}}$, consistent with the expected reduced impact of nuclear parton distributions and coherent energy loss effects for increasing $\mathrm{J}/{\psi }$ $p_{\mathrm {T}}$. At negative $y_{\mathrm {CM}}$, both shadowing and energy loss effects are known to lead to small nuclear modifications, as confirmed by the present measurements. Such processes are also expected to affect the nuclear dependence of ${\mathrm {B}}$ hadron production and thereby, through its decays, nonprompt $\mathrm{J}/{\psi }$ production. The measurements presented here provide new constraints on cold nuclear matter effects on prompt and nonprompt $\mathrm{J}/{\psi }$ production over a wide kinematic range.
